# Comparison of the immunogenicity and safety of Euvichol-Plus with Shanchol in healthy Indian adults and children: an open-label, randomised, multicentre, non-inferiority, parallel-group, phase 3 trial

**DOI:** 10.1016/j.lansea.2023.100256

**Published:** 2023-08-24

**Authors:** Sanket Shah, Ranjan Kumar Nandy, Shaily S. Sethi, Bhakti Chavan, Sarang Pathak, Shanta Dutta, Sanjay Rai, Chandramani Singh, Vinod Chayal, Chintan Patel, N. Ravi Kumar, Abhishek T. Chavan, Amit Chawla, Anit Singh, Anupriya Khare Roy, Nidhi Singh, Yeong Ok Baik, Youngjin Lee, Youngran Park, Kyung Ho Jeong, Syed Ahmed

**Affiliations:** a Techinvention Lifecare Private Limited, Mumbai, India; b Indian Council of Medical Research - National Institute of Cholera and Enteric Diseases, Kolkata, India; c All India Institute of Medical Sciences, New Delhi, India; d All India Institute of Medical Sciences, Patna, India; e Pandit Bhagwat Dayal Sharma Post Graduate Institute of Medical Sciences, Rohtak, India; f Aatman Hospital, Ahmedabad, India; g Niloufer Hospital, Hyderabad, India; h Jeevan Rekha Hospital, Belagavi, India; i Prakhar Hospital, Kanpur, India; j Clinical Research Network India, Noida, India; k EuBiologics Co., Ltd., Seoul, South Korea

**Keywords:** Cholera, Euvichol-Plus, Immunogenicity, India, Non-inferiority, Oral cholera vaccines, Phase 3, Safety, Shanchol

## Abstract

**Background:**

Considering the cholera menace in India and to seek licensure of the oral cholera vaccine (OCV), Euvichol-Plus, we conducted a clinical trial to compare the immunogenicity and safety of Euvichol-Plus with Shanchol in healthy Indian adults and children.

**Methods:**

This phase 3, open-label, multicentre, randomised, non-inferiority, parallel-group, comparative study was conducted at seven sites across India involving 416 healthy adults (aged ≥18–60 years) and children (aged ≥1 to <18 years). Healthy individuals who agreed to participate through a voluntary written informed consent form along with oral or written assent (for children aged 7–18 years) were included. No assent was required for those <7 years, as consent was given by the legally acceptable representatives (LAR). Participants were randomised 1:1 to receive two doses of either Euvichol-Plus or Shanchol orally, 14 days apart. The first dose (1.5 ml) was administered on visit 1, and the second dose at 2 weeks after the first dose during visit 2. Participants were followed up telephonically for 3 consecutive days after each visit and returned for final assessment at 2 weeks after the second dose (visit 3). Blood samples were collected for immunogenicity assessment, and safety analyses were done during all the visits. The primary immunogenicity endpoint was the percentage of participants with ≥4-fold increase in anti-*Vibrio cholerae* (*V. cholerae*) O1 Ogawa and O1 Inaba (vibriocidal) antibody titres at 2 weeks after the second dose as compared to baseline titres prior to dosing. The secondary immunogenicity endpoints included the percentage of participants with ≥4-fold increase in anti-*V. cholerae* O139 antibody titres at 2 weeks after the second dose as compared to baseline titres, and geometric mean titres (GMT) and geometric mean ratios (GMR) as measured by anti-*V. cholerae* O1 Ogawa, O1 Inaba, and O139 antibody titres at 2 weeks after the second dose as compared to baseline titres. The safety endpoints included assessment of solicited, unsolicited adverse events (AEs), and serious adverse events (SAEs). The clinical trial was registered with the Clinical Trials Registry of India (CTRI/2021/08/035344).

**Findings:**

The study was performed in two age cohorts: cohort 1 (aged ≥18–60 years, 208 participants [104 in Euvichol-Plus group and 104 in Shanchol group]), and cohort 2 (aged ≥1 to <18 years, 208 participants [104 in Euvichol-Plus group and 104 in Shanchol group]). A total of 414 participants (Euvichol-Plus: 206 and Shanchol: 208) who completed the study (intention-to-treat and per-protocol set) were analysed to compare the vibriocidal titre as an index for immunogenicity. At 2 weeks after the second dose, the percentage of participants in the Euvichol-Plus group who reported a ≥4-fold increase in anti-*V. cholerae* antibody titres were 68.93% (O1 Ogawa) [95% CI 62.13%–75.18%], 66.02% (O1 Inaba) [95% CI 59.11%–72.46%], and 59.71% (O139) [95% CI 52.67%–66.47%] as compared to 63.94% (O1 Ogawa) [95% CI 57.01%−70.47%], 65.87% (O1 Inaba) [95% CI 58.99%–72.28%], and 56.25% (O139) [95% CI 49.22%–63.10%] in the Shanchol group. The lower limit of 95% CI for treatment difference for all the antibody titres was ≥10% (non-inferiority margin), demonstrating that Euvichol-Plus was non-inferior to Shanchol. The post-vaccination GMT (Day 14 and 28) were more than the pre-vaccination GMT for all three serotypes in both groups. The GMR obtained for Euvichol-Plus over Shanchol for O1 Ogawa, O1 Inaba, and O139 serotypes was >1, indicating non-inferiority of Euvichol-Plus to Shanchol. The safety cohort included 416 participants. Headache was the most common solicited AE, whereas cold and cough were the most common unsolicited AEs in both groups.

**Interpretation:**

Euvichol-Plus appears to be non-inferior to Shanchol in terms of immunogenicity and safety in healthy Indian adults and children.

**Funding:**

Techinvention Lifecare Private Limited, Mumbai, India.

**Research in context**

**Evidence before this study**

In a phase 1 clinical study of Euvichol conducted in 20 healthy adult males in South Korea, 95% participants demonstrated ≥4-fold increase in *Vibrio cholerae* (*V. cholerae*) O1 Inaba and Ogawa antibodies, and 45% demonstrated ≥4-fold increase in *V. cholerae* O139 antibodies following a two-dose schedule given 2 weeks apart. No clinically relevant safety issue was observed. A phase 3 study conducted in the Philippines involving 1263 healthy participants showed that the vibriocidal antibody response to O1 Inaba following administration of two doses of Euvichol was non-inferior to Shanchol in adults (82% vs. 76%) and children (87% vs. 89%). Similar findings were observed for O1 Ogawa in adults (80% vs. 74%) and children (91% vs. 88%). Euvichol received World Health Organization (WHO) prequalification in 2015. Further, a bridging study conducted in 442 healthy adults and children in the Philippines demonstrated the equivalence of the two formulations of Euvichol (100L with thiomersal vs. 600L without thiomersal) with respect to seroconversion rates and vibriocidal antibody response against O1 Inaba, O1 Ogawa, and O139 serotypes. The thiomersal-free Euvichol received WHO prequalification in 2016. To further improve Euvichol, EuBiologics changed the presentation of the vaccine from conventional glass vials to plastic tubes, viz., Euvichol-Plus (thiomersal-free). This improved version offers numerous advantages, received WHO prequalification in 2017, and dominates the global oral cholera vaccine (OCV) stockpile. A matched case–control study involving 79 cases and 316 controls demonstrated that two-dose regimens of Euvichol-Plus conferred effective protection, which was administered during a mass vaccination campaign following a cholera outbreak in Lusaka, Zambia, during 2017–2018. The adjusted odds ratio (AOR) vaccine effectiveness for two doses was 81%, and secondary analysis showed that vaccine effectiveness for any dose (one or more dose) was 74%. India features at the top of the list of cholera-endemic countries and is not supported by Gavi, the Vaccine Alliance, for OCV. The production of Shanchol, which has been the licensed OCV in India, will be discontinued by Sanofi Pasteur in 2023. Euvichol-Plus is not yet licensed in India.

**Added value of this study**

Our study is the first one to compare the immunogenicity and safety of Euvichol-Plus with Shanchol in the Indian population. Euvichol-Plus appears to be non-inferior to Shanchol in terms of immunogenicity and safety in healthy Indian adults and children. It will pave the way for Euvichol-Plus in India and other countries globally where cholera continues to be a significant public health threat.

**Implications of all the available evidence**

The results of our study supports the existing evidence, showing the non-inferiority of Euvichol-Plus to Shanchol. Euvichol-Plus can be used as a suitable alternative to Shanchol in cholera control programmes, especially when Sanofi Pasteur has decided to discontinue the production of Shanchol in 2023.

## Introduction

Cholera continues to be a global public health threat, with 1.3–4.0 million cases and 21,000–143,000 deaths worldwide annually.^1^^,^^2^ There are 1.3 billion people at risk of cholera in endemic countries.^3^ Cholera is a diarrhoeal illness caused by the ingestion of food or water contaminated with toxigenic strains of the bacterium *Vibrio cholerae* (*V. cholerae*) serogroups O1 or O139.^4^ If left untreated, it can result in severe dehydration, hypovolaemic shock, and death.^4^ Cholera has been responsible for large epidemics and even pandemics.^4^ With an estimated incidence rate of 1.64 per 1000, India features at the top of the list of cholera-endemic countries.^5^ In India, estimates show that 400 million people are at risk of cholera, with 6.7 million cases and 20,000 (3%) deaths annually.^6^ Cholera occurs with marked seasonal dynamics in India, being prevalent in the hot, humid, and rainy seasons.^7^ The surveillance data of India shows that there is a steady increase in reported cholera outbreaks throughout the country.^7^ From 2011 to 2020, 565 outbreaks were reported in India that led to 45,759 cases and 263 deaths.^7^ This represents only the tip of the iceberg, as cholera remains an under-recognised health issue and grossly under-reported in India.^7^^,^^8^

The mainstay of cholera control measures is to improve water, sanitation, and hygiene (WaSH) practices. However, vaccination is a key intervention to prevent or mitigate cholera.^9^^,^^10^ A major advance in addressing this persisting global problem was the development and creation of a global stockpile of cheap, safe, and effective inactivated whole-cell oral cholera vaccines (OCVs) by the World Health Organization (WHO) in 2013 to respond to emergency situations.^10^^,^^11^ With expansion in the OCV supply and demonstration of its successful impact in multiple countries, the WHO, through its Global Task Force on Cholera Control (GTFCC), launched the Ending Cholera: Global Roadmap in 2030, with the aim of reducing cholera deaths by 90% through enhanced detection and response to outbreaks, targeted use of OCVs in outbreaks and in preventive campaigns in hotspots, together with integrated improvements in water and sanitisation.^12^ Several studies have been conducted on OCVs to determine their safety, efficacy, effectiveness, field feasibility, and acceptance in high-risk urban populations.^13^ OCVs have been found to be effective in cholera-endemic, epidemic, and outbreak settings.^9^^,^^14^ Vaccine trials conducted in India by the investigators from the National Institute of Cholera and Enteric Diseases under the Indian Council of Medical Research (ICMR-NICED) for whole-cell killed OCV showed a protective efficacy of 65%, almost consistent during 2–5 years post-vaccination.^6^ Currently, there are three WHO prequalified OCVs: Dukoral (Valneva, France), Shanchol (Shantha Biotechnics Ltd., [acquired by Sanofi Pasteur], India), and Euvichol-Plus (EuBiologics Co., Ltd. [EuBiologics], South Korea); the latter two are included in the WHO global OCV stockpile.^9^^,^^15^, ^16^, ^17^

In 2010, the International Vaccines Institute (IVI) initiated a partnership with EuBiologics to facilitate a technology transfer of OCV with an aim to establish additional manufacturers apart from Shanchol (developed through a technology transfer facilitated by IVI between VaBiotech [Vietnam] and Shantha Biotechnics Ltd.) to cater to the increasing global demand. This led to the production of Euvichol, with a manufacturing process and composition identical to Shanchol.^15^, ^16^, ^17^ Euvichol was evaluated for safety and immunogenicity in phase 1 and phase 3 clinical trials in South Korea and the Philippines, respectively.^18^^,^^19^ It received WHO prequalification in 2015.^15^ A bridging study was also conducted in the Philippines to demonstrate the equivalence of thiomersal-free 600L Euvichol with the original Euvichol formulation (100L with thiomersal).^20^ The thiomersal-free Euvichol received WHO prequalification in 2016.^15^ To further improve Euvichol, EuBiologics changed the presentation of the vaccine from conventional glass vials to plastic tubes, viz., Euvichol-Plus (thiomersal-free). With Euvichol-Plus, there has been a reduction in the vial’s volume by 30% and weight by over 50%, offering easier storage, transportation, and distribution, lower overall production costs, ease of administration, and facilitating delivery in emergency situations and humanitarian crisis in campaign mode. This improved version received WHO prequalification in 2017.^9^^,^^15^^,^^16^ The effectiveness of two-dose regimen of Euvichol-Plus has been evaluated in response to the cholera outbreak in Lusaka, Zambia, during 2017–2018.^14^ Euvichol-Plus has not been licensed in India.^12^ Given the occurrence of cholera outbreaks in India and to seek the licensure of Euvichol-Plus, we conducted a phase 3 trial to compare the immunogenicity and safety of Euvichol-Plus with Shanchol in healthy Indian adults and children.

## Methods

### Study design and participants

This was a phase 3, open-label, multicentre, randomised, non-inferiority, parallel-group, comparative clinical study conducted at seven sites across India: All India Institute of Medical Sciences, New Delhi; Pandit Bhagwat Dayal Sharma Post Graduate Institute of Medical Sciences, Rohtak; All India Institute of Medical Sciences, Patna; Aatman Hospital, Ahmedabad; Niloufer Hospital, Hyderabad; Jeevan Rekha Hospital, Belagavi; and Prakhar Hospital, Kanpur. Adults (aged ≥18–60 years) and children (aged ≥1–18 years) considered healthy as per the medical history, physical examination, and clinical judgement of the investigator, as well as those who could be followed up during the study period and could comply with the study requirements, were included in the study. Also, individuals who agreed through a voluntary written informed consent form (signed by parents or legally acceptable representatives [LAR] of participants aged <18 years) along with signed assent (for participants aged 12 to <18 years) or oral assent (for participants aged 7–11 years) were included in the study. However, no assent was required for those <7 years, as consent could be given by the LAR.

Participants with a history of cholera or cholera vaccinations, hypersensitivity reactions to other preventative vaccinations, immune function disorders (including immunodeficiency diseases), 38°C or higher body temperature measured prior to the investigational product (Euvichol-Plus and Shanchol) dosing, or experiencing diarrhoea or abdominal pain lasting 2 weeks or longer within 6 months prior to study initiation were excluded. Also, participants requiring administration of anti-diarrhoeal drugs or antibiotics to treat diarrhoea within one week prior to study initiation or experiencing abdominal pain, nausea, vomiting, or decreased appetite within 24 hours prior to study trial initiation were excluded. Moreover, pregnant or lactating women, children vaccinated within one month prior to study initiation or planned vaccination during the study, participants in another clinical trial with investigational product dosing within 6 months prior to study initiation, or if any of the individuals having difficulty in participating in the study due to severe chronic diseases or due to other reasons (based on the judgement of the investigator), and female patients getting pregnant after the first dose, were excluded.

Participants were enrolled in the study in descending order of age. Healthy adults aged ≥18–60 years were enrolled initially as cohort 1, and the study vaccines were administered. The 14-day post second dose safety data of the participants in cohort 1 were reviewed by the Data and Safety Monitoring Board (DSMB) before proceeding with the enrolment of participants for cohort 2 (healthy children aged ≥1 to <18 years).

The study was performed in accordance with the current version of the Declaration of Helsinki (64^th^ World Medical Association General Assembly, Fortaleza, Brazil, October 2013). The study was performed in compliance with the study protocol, the Indian Council of Medical Research (ICMR) guidelines on Biomedical Research on Children (2017), current version of the Central Drugs Standard Control Organization (CDSCO)–Good Clinical Practice (GCP) guidelines, and the New Drugs and Clinical Trials Rules, 2019. The study protocol, informed consent, and other information that required pre-approval were reviewed and approved by the respective Institutional Ethics Committees (IEC). The clinical trial was registered with the Clinical Trials Registry of India (CTRI/2021/08/035344).

### Randomisation

Randomisation was employed as an unbiased method of assigning the participants to the immunogenicity group. A centralized randomisation list for the study was prepared by the independent biostatistician to prevent bias. All sites were provided sealed randomisation envelopes containing study arm detail by the independent biostatistician. Participants meeting the inclusion and exclusion criteria were enrolled and allocated to one of the study arms as per randomisation envelopes. Participants enrolled in cohorts 1 and 2, each comprising 208 healthy participants, were randomised and allocated in a 1:1 ratio to receive either Euvichol-Plus (total 208 participants with 104 participants in each cohort) or Shanchol (total 208 participants with 104 participants in each cohort).

### Procedures

Each 1.5 ml oral dose of the comparator vaccine, Shanchol (Sanofi Pasteur India Private Limited; Batch number: SCN015A19; expiry date: 11/2021), contained *V. cholerae* O1 Inaba E1 Tor strain Phil 6973 formaldehyde killed (600 ELISA units [EU] of lipopolysaccharide [LPS]), *V. cholerae* O1 Ogawa classical strain Cairo 50 heat killed (300 EU of LPS), *V. cholerae* O1 Ogawa classical strain Cairo 50 formaldehyde killed (300 EU of LPS), *V. cholerae* O1 Inaba classical strain Cairo 48 heat killed (300 EU of LPS), and *V. cholerae* O139 strain 4260B formaldehyde killed (600 EU of LPS). The vaccine was available as a single dose vial.

Each 1.5 ml oral dose of the test vaccine, Euvichol-Plus (EuBiologics Co., Ltd.; Batch number: EP21023; expiry date: March 11, 2023), contained *V. cholerae* O1 Inaba Cairo 48 classical biotype heat inactivated (300 Lipopolysaccharide ELISA units [L.E.U]), *V. cholerae* O1 Inaba Phil 6973 El Tor biotype formalin inactivated (600 L.E.U), *V. cholerae* O1 Ogawa Cairo 50 classical biotype formalin inactivated (300 L.E.U), *V. cholerae* O1 Ogawa Cairo 50 classical biotype heat inactivated (300 L.E.U), and *V. cholerae* O139 4260 B formalin inactivated (600 L.E.U). The vaccine was available as a single dose plastic tube. Both Shanchol and Euvichol-Plus were stored under controlled conditions at 2–8°C before administration.

According to the pre-generated table, the participants were administered their first dose (1.5 ml) of either the test or comparator vaccine orally on visit 1, and follow-up (for safety analyses) was done telephonically for 3 consecutive days. The second dose was administered 2 weeks after the first dose during visit 2. The participants were followed up telephonically for 3 consecutive days after vaccination and returned for final assessment 2 weeks after the second dose (visit 3). Blood samples were withdrawn for immunogenicity assessment at visit 1 (prior to the first dose), visit 2 (prior to the second dose), and visit 3 (2 weeks after the second dose).

Safety analyses were done at all the visits and telephonically for 3 consecutive days after each vaccination. The participants and/or parents/LAR were instructed to record adverse events (AEs) that occurred after the administration of the study vaccines. The study was initiated in October 2021 and completed in December 2021. The participants were included in the study for 28 days.

### Outcomes

The primary immunogenicity endpoint was the assessment of seroconversion rate, viz., the percentage of participants with ≥4-fold increase in anti-*V. cholerae* O1 Ogawa and O1 Inaba antibody titres at 2 weeks after the second dose (visit 2) as compared to baseline titres prior to investigational product dosing (visit 1). The secondary immunogenicity endpoints included the proportion of participants with ≥4-fold increase in anti-*V. cholerae* O139 antibody titres at 2 weeks after the second dose (visit 2) as compared to baseline titres prior to investigational product dosing (visit 1); geometric mean titres (GMT) and geometric mean ratio (GMR) as measured by anti-*V. cholerae* O1 Ogawa and O1 Inaba (vibriocidal) antibody titres at 2 weeks after the second dose (visit 2) as compared to baseline titres prior to investigational product dosing (visit 1); and GMT and GMR as measured by anti-*V. cholerae* O139 antibody titres at 2 weeks after the second dose (visit 2) as compared to baseline titres prior to investigational product dosing (visit 1). Serum vibriocidal titres were reported using GMT and geometric mean fold-rise (GMFR).

The safety endpoints (from enrolment until the end of the study period) included solicited AEs (Day 0–3) post each vaccine dose and unsolicited AEs, including abnormal vital signs and physical examination throughout the study. Serious adverse events (SAEs), including abnormal vital signs and physical examination throughout the study, were also part of the safety endpoints.

### Statistical analysis

As per published clinical study evaluating the immunogenicity of Shanchol and Euvichol, the seroconversion rates (4-fold rise in antibody titres), 14 days after the first and second dose of the vaccine ranges from 73% to 90% in adults and children.^19^ Assuming a seroconversion rate of 70% in the comparator group, power of 90%, alpha of 5% (two-sided), and a non-inferiority margin of 10%, 186 participants would be required in the test and comparator groups to establish the non-inferiority of the test vaccine. Considering a dropout rate of 10%, a total of 416 participants were enrolled in the study (208 in the test vaccine group and 208 in the comparator vaccine group).

All eligible participants were studied for analyses of demographics and baseline statistics. The safety set included all randomised participants who had taken at least one dose of the investigational product, based on which safety was assessed. The intention-to-treat (ITT) set included all randomised participants who had at least one measurement of anti-*V. cholerae* O1 Ogawa, O1 Inaba, and O139 vibriocidal antibody titres after investigational product dosing, based on which immunogenicity was assessed. The per-protocol (PP) set included ITT participants who completed the study per-protocol, based on which immunogenicity was assessed additionally.

Demographic characteristics (age, sex, gender, height, weight, and body mass index [BMI]) of each study cohort were tabulated. Continuous variables were summarised using mean, standard deviation, median, and range (minimum and maximum), while categorical variables were summarized using proportions (counts and percentages). The p-value for comparing continuous variables like GMR, GMT, and GMFR was computed using the Student’s *t*-test. The p-value for comparing categorical variables, viz., adverse events and seroconversion rates, was computed using Chi-square test. For primary immunogenicity endpoint analysis, establishing non-inferiority of Euvichol-Plus with Shanchol in terms of percentage of participants with at least four times higher anti-*V. cholerae* O1 Ogawa and Inaba antibody titres at 2 weeks after the second dose, 95% CI on the difference in proportions were computed using two-group pooled Z-test. Noninferiority was declared as the lower bound of the two-sided 95% CI for the treatment difference (Euvichol-Plus–Shanchol) in proportions was ≥−10%. All statistical analyses were done as per p-values based on 2-sided tests, and p-values <0.05 were considered to be statistically significant. XLSTAT version 2021.3.1 and R version 4.0.5 were used for statistical analyses.

### Role of the funding source

The funder of the study had no role in the study design, data collection, data analyses, data interpretation, or writing of the report. Fund provided by Techinvention Lifecare Private Limited was received by ICMR-NICED for carrying out bioanalytic testing at ICMR-NICED.

## Results

This clinical study was conducted in 416 healthy participants (adults and children) in the age group of ≥1–60 years, stratified into two age cohorts shown below:

• Cohort 1 [aged ≥18–60 years, 208 participants (104 in Euvichol-Plus group and 104 in Shanchol group)]

• Cohort 2 [aged ≥1 to <18 years, 208 participants (104 in Euvichol-Plus group and 104 in Shanchol group)]

A summary of baseline demographic characteristics for the participants is presented in Table 1. All baseline demographics were calculated per safety set (all participants who had at least one dose of the investigational product). The average age, height, weight, and BMI of total study participants (n = 416) were 23.8 ± 15.61 years (1–60 years), 144.2 ± 28.31 cm (46–184 cm), 47.6 ± 20.09 kg (7–90 kg), and 21.8 ± 4.84 kg/m^2^ (10.59–42.2 kg/m^2^), respectively. Overall, the demographic characteristics of the participants recorded at the screening visit were similar between the Euvichol-Plus and the Shanchol groups. The average age of participants who received Euvichol-Plus was 24.0 ± 15.81 years, while that of participants receiving Shanchol was 23.6 ± 15.44 years. Similarly, the average weight (Euvichol-Plus: 47.6 ± 20.34 kg, Shanchol: 47.5 ± 19.88 kg), height (Euvichol-Plus: 144.2 ± 28.71 cm, Shanchol: 144.2 ± 27.98 cm), and BMI (Euvichol-Plus: 21.7 ± 4.6 kg/m^2^, Shanchol: 21.9 ± 5.08 kg/m^2^) of participants in both groups were found to be comparable.

**Table 1:** Demographic characteristics of the study participants.

Parameter	Categories	Euvichol-Plus (N = 208)	Shanchol (N = 208)	Total (N = 416)
Age (Years)	Mean	24	23.6	23.8
	SD	15.81	15.44	15.61
Gender	Male	106 (51%)	114 (54.8%)	220 (52.9%)
	Female	102 (49%)	94 (45.2%)	196 (47.1%)
Height (cm)	Mean	144.2	144.2	144.2
	SD	28.71	27.98	28.31
Weight (kg)	Mean	47.6	47.5	47.6
	SD	20.34	19.88	20.09
BMI (kg/m^2^)	Mean	21.7	21.9	21.8
	SD	4.60	5.08	4.84

BMI: Body mass index, N: Number of participants, SD: Standard deviation. Percentages were calculated using the respective column header count as denominator.

Out of 416 total study participants, 52.9% (220/416) were males and 47.1% (196/416) were females. In the Euvichol-Plus group, there were 106/208 (51%) male participants and 102/208 (54.8%) were female participants, whereas in the Shanchol group, 114/208 (54.8%) were male participants and 94/208 (45.2%) were female participants.

**Fig. 1:**
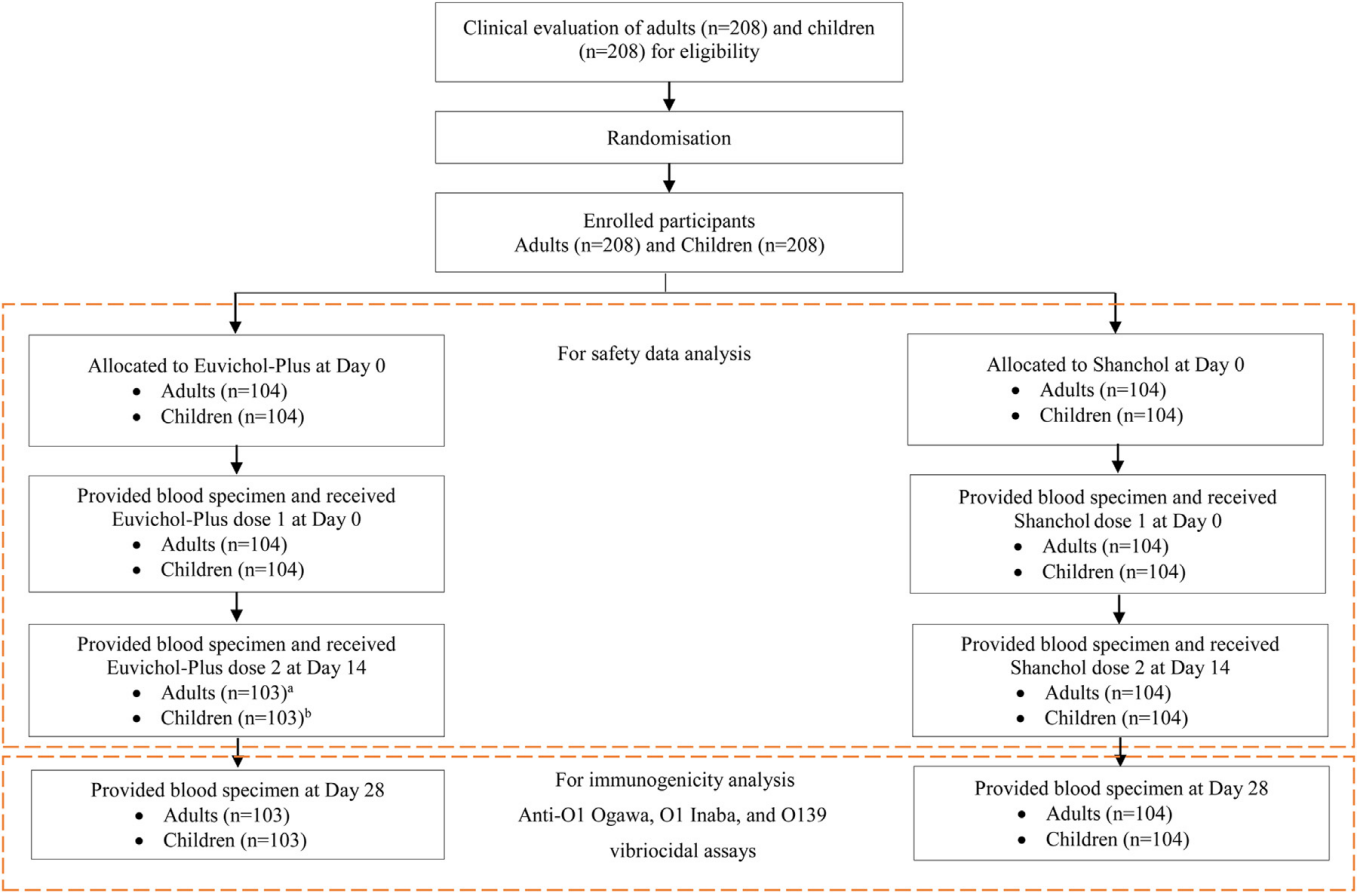
**Study design flowchart (cohort 1 and cohort 2)∗.** ∗The enrolment for cohort 1 and cohort 2 was done sequentially or in stepwise manner. Note: (a) 1 participant died; (b) 1 participant lost to follow-up.

The study design flowchart is shown in Fig. 1. A total of 414 participants (206 in the Euvichol-Plus group and 208 in the Shanchol group) who completed the study (ITT and PP set) were analysed to compute and compare the immunogenicity of Euvichol-Plus with Shanchol in healthy Indian adults and children.

**Fig. 2:**
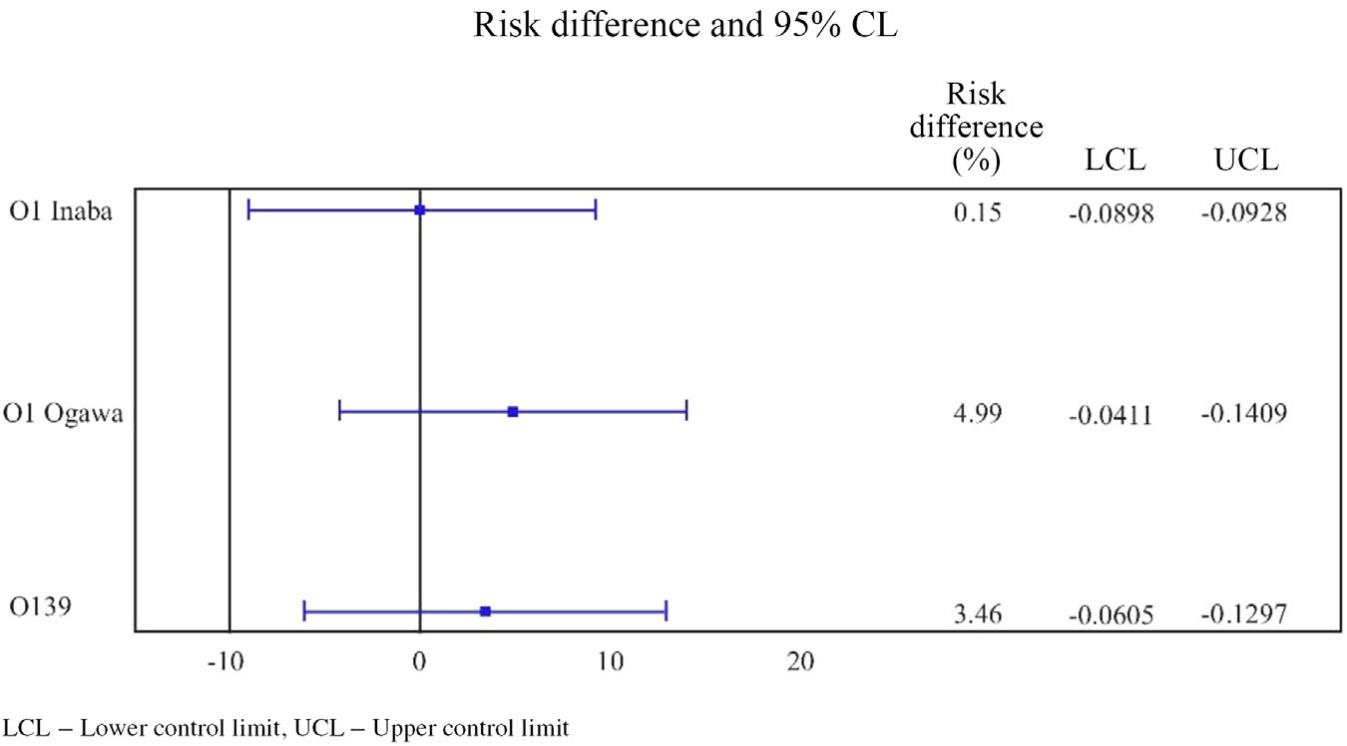
**Forest plot for participants with ≥4-foldincrease in anti-*****V. cholerae*****O1 Ogawa, O1 Inaba, and O139 antibody titres at 2 weeks after the second dose.**

Table 2 highlights the percentage of participants exhibiting ≥4-fold increase in anti-*V. cholerae* antibody titres for O1 Inaba, O1 Ogawa, and O139 at 2 weeks after the first and second dose of the vaccines, as well as the differences between the seroprotection rates along with the associated 95% CI for both groups. In the Euvichol-Plus group, the percentage of participants who attained ≥4-fold increase in anti-*V. cholerae* antibody titres for O1 Ogawa, O1 Inaba, and O139 serotypes at 2 weeks after the first dose were 77.18% (95 CI 70.84%–82.73%), 73.3% (95% CI 66.71%−79.21%), and 60.19% (95 CI 53.16%–66.93%), respectively; in the Shanchol group, the same were 71.15% (95% CI 64.48%–77.21%), 75% (95% CI 68.54%–80.73%), and 59.62% (95% CI 52.61%–66.34%), respectively. The percentage of participants who reported ≥4-fold increase in anti-*V. cholerae* (vibriocidal) antibody titres at 2 weeks after the second dose in the Euvichol-Plus group were 68.93% (O1 Ogawa) [95% CI 62.13%–75.18%], 66.02% (O1 Inaba) [95% CI 59.11%–72.46%], and 59.71% (O139) [95% CI 52.67%–66.47%] as compared to 63.94% (O1 Ogawa) [95% CI 57.01%−70.47%], 65.87% (O1 Inaba) [95% CI 58.99%–72.28%], and 56.25% (O139) [95% CI 49.22%–63.10%] in the Shanchol group. Notably, the lower limit of 95% CI for treatment difference for all the antibody titres was ≥10% (non-inferiority margin), demonstrating that Euvichol-Plus was non-inferior to Shanchol as per the study endpoints. Of the 206 participants in the Euvichol-Plus arm, 151, 159, and 124 participants showed seroconversion against O1 Inaba, O1 Ogawa, and O139, respectively, at 2 weeks following the first dose. Of the 208 participants in the Shanchol arm, seroconverted participants were 156, 148, and 124 against O1 Inaba, O1 Ogawa, and O139, respectively. There was a slight reduction in the number of seroconverted participants in either the Euvichol-Plus or the Shanchol arm at 2 weeks after the second dose. At 2 weeks following the second dose, seroconverted participants among the Euvichol-Plus arm were 136, 142, and 123 vs. 137, 133, and 117 among the Shanchol arm against O1 Inaba, O1 Ogawa, and O139, respectively. The Forest plot for participants with ≥4-fold increase in anti-*V. cholerae* O1 Ogawa, O1 Inaba, and O139 antibody titres at 2 weeks after the second dose is shown in Fig. 2.

**Table 2:** Percentage of participants with ≥4-fold increase in *anti-V. cholerae* O1 Ogawa, O1 Inaba, and O139 antibody titres and differences in seroprotection rates between groups at 2 weeks after the first dose and second dose of Euvichol-Plus and Shanchol.

Percentage of participants with ≥4-fold increase in anti-*V. cholerae* O1 Ogawa, O1 Inaba, and O139 antibody titres and differences in seroprotection rates between groups at 2 weeks after the first dose				
Antibody	Euvichol-Plus (N = 206), n (%), [95% CI]	Shanchol (N = 208), n (%), [95% CI]	Difference in proportions between groups, [95% CI]	p-value
anti-*V. cholerae* O1 Ogawa	159 (77.18%), [70.84, 82.73]	148 (71.15%), [64.48, 77.21]	6.03, [−2.38, 14.44]	0.1611
anti-*V. cholerae* O1 Inaba	151 (73.30%), [66.71, 79.21]	156 (75.00%), [68.54, 80.73]	−1.70, [−10.13, 6.73]	0.6930
anti-*V. cholerae* O139	124 (60.19%), [53.16, 66.93]	124 (59.62%), [52.61, 66.34]	0.58, [−8.86, 10.02]	0.9044

Percentage of participants with ≥4-fold increase in anti-*V. cholerae* O1 Ogawa, O1 Inaba, and O139 antibody titres and differences in seroprotection rates between groups at 2 weeks after the second dose				
Antibody	Euvichol-Plus (N = 206), n (%), [95% CI]	Shanchol (N = 208), n (%), [95% CI]	Difference in proportions between groups [95% CI]	p-value
anti-*V. cholerae* O1 Ogawa	142 (68.93%), [62.13, 75.18]	133 (63.94%), [57.01, 70.47]	4.99, [−4.09, 14.07]	0.2824
anti-*V. cholerae* O1 Inaba	136 (66.02%), [59.11, 72.46]	137 (65.87%), [58.99, 72.28]	0.15, [−8.98, 9.28]	0.9736
anti-*V. cholerae* O139	123 (59.71%), [52.67, 66.47]	117 (56.25%), [49.22, 63.10]	3.46, [−6.04, 12.96]	0.4759

Percentages were calculated using the respective column header count as denominator. 95% CI was calculated by Clopper-Pearson method. Two-sided 95% CI for difference in proportion of participants between groups was calculated using pooled Z-test. p-value was calculated using Chi-square test.

Antibody GMT (along with 95% CI) at baseline (Day 0), 2 weeks after the first dose (Day 14), and 2 weeks after the second dose (Day 28) are presented in Table 3. The post-vaccination GMT (Day 14 and Day 28) were more than the pre-vaccination GMT for all three serotypes. It is evident from Table 3 that baseline (Day 0) vibriocidal GMT were 12.16 and 12.80 against *V. cholerae* O1 Inaba for the participants who received either Euvichol-Plus or Shanchol, respectively. Baseline GMT values against *V. cholerae* O1 Ogawa were 9.87 and 11.09 in the case of the Euvichol-Plus or the Shanchol recipient groups, respectively. Vibriocidal GMT values determined against O139 were 21.28 and 18.22 from the participants of the Euvichol-Plus or the Shanchol groups, respectively. All these showed that the baseline vibriocidal GMT were comparable among the participants who received either Euvichol-Plus or Shanchol, irrespective of the nature of antibodies directed to either *V. cholerae* O1 Inaba, O1 Ogawa, or O139. Oral administration of either Euvichol-Plus or Shanchol caused a rise in vibriocidal titres among participants. In fact, vibriocidal GMT increased to 157.86, 109.03, and 161.60 from the baseline against O1 Inaba, O1 Ogawa, and O139, respectively, among participants of the Euvichol-Plus arm after 2 weeks following the first dose. The rise of vibriocidal GMT among the Shanchol recipients were 149.68, 113.14, and 138.86 against O1 Inaba, O1 Ogawa, and O139, respectively, which were comparable to the Euvichol-Plus arm. The rise of vibriocidal GMT remained comparable among the participants of the Euvichol-Plus or the Shanchol arms after 2 weeks following the second dose of the respective vaccine. However, the snubbing of immune response after 2 weeks following the second dose was observed, a scenario already reported earlier for OCV. These data showed that immune induction remained similar among the participants who received either Euvichol-Plus or Shanchol, both for the first and second dose.

**Table 3:** Antibody geometric mean titres (GMT) at 2 weeks after the first and second dose of Euvichol-Plus and Shanchol.

Antibody GMT at 2 weeks after the first dose							
Parameter	Visit	Treatment	N	Geometric mean	Lower limit	Upper limit	p-value
O1 Inaba	Day 0	Euvichol-Plus	206	12.16	9.93	14.88	0.7655
		Shanchol	208	12.80	10.47	15.64	
	Day 14	Euvichol-Plus	206	157.86	134.71	184.99	0.6996
		Shanchol	208	149.68	127.15	176.21	
O1 Ogawa	Day 0	Euvichol-Plus	206	9.87	8.26	11.79	0.4500
		Shanchol	208	11.09	9.24	13.31	
	Day 14	Euvichol-Plus	206	109.03	92.37	128.69	0.8023
		Shanchol	208	113.14	94.59	135.32	
O139	Day 0	Euvichol-Plus	206	21.28	17.22	26.32	0.3785
		Shanchol	208	18.22	14.93	22.24	
	Day 14	Euvichol-Plus	206	161.60	134.60	194.01	0.3165
		Shanchol	208	138.86	117.11	164.65	

Antibody GMT at 2 weeks after the second dose							
Parameter	Visit	Treatment	N	Geometric mean	Lower limit	Upper limit	p-value
O1 Inaba	Day 0	Euvichol-Plus	206	12.16	9.93	14.88	0.7655
		Shanchol	208	12.80	10.47	15.64	
	Day 28	Euvichol-Plus	206	90.30	77.81	104.80	0.2209
		Shanchol	208	77.12	66.26	89.76	
O1 Ogawa	Day 0	Euvichol-Plus	206	9.87	8.26	11.79	0.4500
		Shanchol	208	11.09	9.24	13.31	
	Day 28	Euvichol-Plus	206	68.99	59.77	79.63	0.6050
		Shanchol	208	64.63	55.59	75.15	
O139	Day 0	Euvichol-Plus	206	21.28	17.22	26.32	0.3785
		Shanchol	208	18.22	14.93	22.24	
	Day 28	Euvichol-Plus	206	142.94	119.49	171.00	0.0833
		Shanchol	208	110.83	94.15	130.46	

Student’s *t*-test was performed to test significance between the groups.

The GMFR of anti-*V. cholerae* antibody titres (along with 95% CI) at 2 weeks after the first and second dose of the vaccines is presented in Table 4. The GMFR at 2 weeks after the first dose in the Euvichol-Plus group was 12.99 (O1 Inaba), 11.05 (O1 Ogawa), and 7.59 (O139) as compared to 11.69 (O1 Inaba), 10.20 (O1 Ogawa), and 7.62 (O139) in the Shanchol group. The GMFR at 2 weeks after the second dose in the Euvichol-Plus group was 7.43 (O1 Inaba), 6.99 (O1 Ogawa), and 6.71 (O139) as compared to 6.03 (O1 Inaba), 5.83 (O1 Ogawa), and 6.08 (O139) in the Shanchol group. There was a reduction in the GMFR after the second dose as compared to the first dose, as observed in both groups. The GMFR of anti-*V. cholerae* antibody titres (along with 95% CI) at 2 weeks after the first and second dose of the vaccines in children (age groups 1–5 years and 6–17 years) is presented in Table 5. In children (age group 1–5 years), the GMFR at 2 weeks after the first dose in the Euvichol-Plus group was 15.13 (O1 Inaba), 10.70 (O1 Ogawa), and 10.38 (O139) as compared to 11.55 (O1 Inaba), 13.59 (O1 Ogawa), and 6.40 (O139) in the Shanchol group. The GMFR at 2 weeks after the second dose in the Euvichol-Plus group was 10.08 (O1 Inaba), 6.19 (O1 Ogawa), and 13.51 (O139) as compared to 4.71 (O1 Inaba), 7.37 (O1 Ogawa), and 3.74 (O139) in the Shanchol group. In children (age group 6–17 years), the GMFR at 2 weeks after the first dose in the Euvichol-Plus group was 15.46 (O1 Inaba), 14.79 (O1 Ogawa), and 7.88 (O139) as compared to 12.39 (O1 Inaba), 13.12 (O1 Ogawa), and 7.43 (O139) in the Shanchol group. The GMFR at 2 weeks after the second dose in the Euvichol-Plus group was 8.50 (O1 Inaba), 9.37 (O1 Ogawa), and 7.05 (O139) as compared to 6.06 (O1 Inaba), 6.65 (O1 Ogawa), and 6.89 (O139) in the Shanchol group. The GMFR was comparable in both groups for all three serotypes after the first and second dose, as reflected in Table 4, Table 5. As shown in Table 6, the GMR obtained for Euvichol-Plus over Shanchol was >1 for O1 Inaba, O1 Ogawa, and O139, indicating that Euvichol-Plus was non-inferior to Shanchol.

**Table 4:** Geometric mean fold-rise (GMFR) at 2 weeks after the first and second dose of Euvichol-Plus and Shanchol.

GMFR at 2 weeks after the first dose						
Parameter	Treatment	N	Geometric mean	Lower limit	Upper limit	p-value
O1 Inaba	Euvichol-Plus	206	12.99	10.43	16.18	0.5591
	Shanchol	208	11.69	9.57	14.29	
O1 Ogawa	Euvichol-Plus	206	11.05	8.99	13.60	0.6465
	Shanchol	208	10.20	8.35	12.47	
O139	Euvichol-Plus	206	7.59	6.14	9.39	0.9845
	Shanchol	208	7.62	6.15	9.45	

GMFR at 2 weeks after the second dose						
Parameter	Treatment	N	Geometric mean	Lower limit	Upper limit	p-value
O1 Inaba	Euvichol-Plus	206	7.43	6.05	9.12	0.2075
	Shanchol	208	6.03	5.03	7.22	
O1 Ogawa	Euvichol-Plus	206	6.99	5.76	8.49	0.2500
	Shanchol	208	5.83	4.89	6.95	
O139	Euvichol-Plus	206	6.71	5.45	8.27	0.5715
	Shanchol	208	6.08	4.98	7.43	

GMFR = Day 14/Day 0.

GMFR = Day 28/Day 0.

Student’s *t*-test was performed to test significance between the groups.

**Table 5:** Geometric mean fold-rise (GMFR) at 2 weeks after the first and second dose of Euvichol-Plus and Shanchol in children (age groups 1–5 and 6–17 years).

GMFR at 2 weeks after the first dose							
Age group	Parameter	Treatment	N	Geometric mean	Lower limit	Upper limit	p-value
1–5	O1 Inaba	Euvichol-Plus	24	15.13	7.96	28.74	0.6240
		Shanchol	17	11.55	5.98	22.28	
	O1 Ogawa	Euvichol-Plus	24	10.70	5.30	21.60	0.7038
		Shanchol	17	13.59	6.02	30.68	
	O139	Euvichol-Plus	24	10.38	5.13	21.01	0.4249
		Shanchol	17	6.40	3.11	13.16	
6–17	O1 Inaba	Euvichol-Plus	79	15.46	10.70	22.33	0.4523
		Shanchol	87	12.39	8.97	17.12	
	O1 Ogawa	Euvichol-Plus	79	14.79	10.29	21.24	0.6842
		Shanchol	87	13.12	9.43	18.24	
	O139	Euvichol-Plus	79	7.88	5.50	11.29	0.8425
		Shanchol	87	7.43	5.27	10.46	

GMFR at 2 weeks after the second dose							
Age group	Parameter	Treatment	N	Geometric mean	Lower limit	Upper limit	p-value
1–5	O1 Inaba	Euvichol-Plus	24	10.08	5.39	18.85	0.1878
		Shanchol	17	4.71	2.20	10.06	
	O1 Ogawa	Euvichol-Plus	24	6.19	2.82	13.54	0.7867
		Shanchol	17	7.37	3.60	15.12	
	O139	Euvichol-Plus	24	13.51	6.78	26.94	0.0225
		Shanchol	17	3.74	2.22	6.28	
6–17	O1 Inaba	Euvichol-Plus	79	8.50	5.97	12.10	0.2149
		Shanchol	87	6.06	4.54	8.08	
	O1 Ogawa	Euvichol-Plus	79	9.37	6.83	12.85	0.1971
		Shanchol	87	6.65	4.90	9.03	
	O139	Euvichol-Plus	79	7.05	4.98	9.99	0.9370
		Shanchol	87	6.89	4.95	9.60	

GMFR = Day 14/Day 0.

GMFR = Day 28/Day 0.

Student’s *t*-test was performed to test significance between the groups.

**Table 6:** Geometric mean ratio (GMR) at 2 weeks after the second dose.

GMR (Euvichol-Plus/Shanchol)			
Antibody	Geometric mean ratio	Lower limit	Upper limit
anti-*V. cholerae* O1 Ogawa	1.07	0.833	1.367
anti-*V. cholerae* O1 Inaba	1.17	0.909	1.508
anti-*V. cholerae* O1 O139	1.29	0.967	1.720

The safety cohort included 416 participants. As shown in Table 7, the total number and percentage of participants in both groups reporting any AEs (solicited or unsolicited, local or systemic) during the study were 25/416 (6%). While 16 participants (3.84%) reported a total of 22 AEs following the first dose, 9 participants (2.16%) reported a total of 12 AEs after the second dose of the test or comparator vaccine. Headache was the most common solicited AE in both groups (3 participants each in both groups). The other AEs reported included fever (2 participants each in both groups), diarrhoea (Euvichol-Plus: 2/208, 1%; Shanchol: 1/208, 0.5%), and nausea/vomiting (Euvichol-Plus: 2/208, 1%; Shanchol: 1/208, 0.5%). Malaise was reported by 1 participant each in both groups. Amongst the unsolicited AEs, the most common were cold (Euvichol-Plus: 3/208, 1.4%; Shanchol: 1/208, 0.5%) and cough (Euvichol-Plus: 3/208, 1.4%; Shanchol: 1/208, 0.5%), followed by abdominal pain (1 participant each in both groups) and body ache (1 participant each in both groups). Weakness and dizziness were reported by 2 participants in the Shanchol group only. All solicited and unsolicited AEs reported after the first and second dose of vaccines were mild in severity, except 3 AEs in the Shanchol group, which were moderate in severity and were completely resolved within 3 days of onset. No participant was withdrawn from the study due to AEs. One serious adverse event (SAE) was reported during the course of the study, which was found as ‘not related’ to causality with the interventional product.

**Table 7:** Summary of adverse events (AEs).

Category	AEs	Euvichol-Plus (N = 208), n (%)	Shanchol (N = 208), n (%)	Total (N = 416), n (%)	p-value
Solicited AEs	Diarrhoea	2 (1.0%)	1 (0.5%)	3 (0.7%)	0.559
	Fever	2 (1.0%)	2 (1.0%)	4 (1.0%)	>0.999
	Headache	3 (1.4%)	3 (1.4%)	6 (1.4%)	>0.999
	Malaise	1 (0.5%)	1 (0.5%)	2 (0.5%)	>0.999
	Nausea/Vomiting	2 (1.0%)	1 (0.5%)	3 (0.7%)	0.559
Unsolicited AEs	Weakness	0 (0.0%)	2 (1.0%)	2 (0.5%)	0.095
	Abdominal pain	1 (0.5%)	1 (0.5%)	2 (0.5%)	>0.999
	Body Ache	1 (0.5%)	1 (0.5%)	2 (0.5%)	>0.999
	Cold	3 (1.4%)	1 (0.5%)	4 (1.0%)	0.304
	Cough	3 (1.4%)	1 (0.5%)	4 (1.0%)	0.304
	Dizziness	0 (0.0%)	2 (1.0%)	2 (0.5%)	0.095

Category	Euvichol-Plus (N = 208), n	Shanchol (N = 208), n	Total (N = 416), n
Total AEs	18	16	34
After first dose	10	12	22
After second dose	8	4	12

Category	Euvichol-Plus (N = 208), n (%)	Shanchol (N = 208), n (%)	Total (N = 416), n (%)
Participants reporting AEs	13 (6.25%)	12 (5.77%)	25 (6%)
After first dose	7 (3.36%)	9 (4.32%)	16 (3.84%)
After second dose	6 (2.89%)	3 (1.45%)	9 (2.16%)

Percentages were calculated using the respective column header group count as denominator. p-value was calculated using Chi-square test.

## Discussion

Our study is the first one comparing the immunogenicity and safety of two prequalified OCVs, Euvichol-Plus with Shanchol, in healthy Indian population. The study suggested that Euvichol-Plus is non-inferior to Shanchol with respect to immunogenicity and safety. Euvichol-Plus is supplied to low- and middle-income countries (LMICs) that are cholera-endemic or regularly experience outbreaks through the WHO stockpile funded by Gavi, the Vaccine Alliance, after receiving WHO prequalification.^21^ It is currently the dominating OCV that is used in the global cholera vaccine stockpile.^17^

In a phase 1 clinical study of Euvichol conducted in 20 healthy adult males in South Korea, no clinically relevant safety issue was observed, and 95% (19/20) participants demonstrated ≥4-fold increase in *V. cholerae* O1 Inaba and Ogawa antibodies, and 45% (9/20) demonstrated ≥4-fold increase in *V. cholerae* O139 antibodies following a two-dose schedule given 2 weeks apart.^18^ A phase 3 study conducted in the Philippines involving 1263 healthy participants (777 adults and 486 children) showed that the vibriocidal antibody response to O1 Inaba following administration of two doses of Euvichol was non-inferior to Shanchol in adults (82% vs. 76%) and children (87% vs. 89%). Similar findings were observed for O1 Ogawa in adults (80% vs. 74%) and children (91% vs. 88%).^19^ Non-inferiority for O139 was inconclusive since 95% CI lower bound value was below the clinical margin (<−10%).^19^ A bridging study conducted in 442 healthy adults and children in the Philippines comparing the two formulations of Euvichol (100L with thiomersal vs. 600L without thiomersal) showed non-inferiority of Euvichol (thiomersal-free) to original Euvichol (with thiomersal) with respect to seroconversion rates and vibriocidal antibody response against O1 Inaba, O1 Ogawa, and O139 serotypes.^20^ In our study, we observed that the percentage of participants who attained ≥4-fold increase in anti-*V. cholerae* antibody titres against O1 Ogawa, O1 Inaba, and O139 serotypes in the Euvichol-Plus group were non-inferior to the Shanchol group, subsequent to the first as well as the second dose of either vaccine. Seroconversion rates in Euvichol-Plus were non-inferior to Shanchol against all three serotypes, as reflected in our study.

We also observed that the seroconversion rates were higher for O1 Inaba and O1 Ogawa as compared to O139 across both groups, which is in line with the previously published studies.^18^, ^19^, ^20^^,^^22^^,^^23^ Plausible explanation for this lower immune response to O139 may be due to the lower antigenic content in the vaccine.^18^^,^^22^^,^^23^ The sensitivity of the assay used may be another factor. The highly diluted complement used in the vibriocidal assay for *V. cholerae* O1 may not be sufficient to mediate the killing of O139, which has a capsule.^22^ *V. cholerae* O139 possesses both lipopolysaccharide (LPS) and capsular polysaccharide (CPS) in the ratio 1:2, with LPS being the minor component.^18^ Higher seroconversion rates and GMT were observed for all three serotypes in both groups after the first dose as compared to the second dose of Shanchol and Euvichol-Plus. This is consistent with the previously published data of Shanchol.^22^^,^^23^ A similar trend was seen in a phase 3 non-inferiority trial conducted by Baik and colleagues for all three serotypes in adult participants (≥18 years) in both Euvichol and Shanchol groups.^19^ However, in children (1–17 years), higher seroconversion rates and GMT were observed only for O139 in both groups.^19^ The higher vibriocidal response after the first dose as compared to the second dose could be due to the high antigenic LPS content, which is about 2-fold higher than the earlier generation of OCV.^22^ Also, the first dose of the vaccine induces an immune response in the intestinal mucosa that inhibits the response to the second dose, and thus the observed lower levels after the second dose may be due to the continued waning of the antibodies.^18^^,^^22^^,^^23^ As high vibriocidal antibody titres are obtained after the first dose, further increase may not be possible after the second dose.^18^^,^^23^

We observed that the GMFR (combined data of adults and children) was higher after the first dose as compared to the second dose for all three serotypes in both Euvichol-Plus and Shanchol groups. A similar pattern was observed in previously published studies of Shanchol.^22^^,^^23^ In a phase 3 non-inferiority trial conducted by Baik and colleagues, a similar trend was observed for all three serotypes in adult participants (≥18 years) in both Euvichol and Shanchol groups. However, in children (1–17 years), similar pattern was observed for O139 in both groups and O1 Inaba in the Euvichol group.^19^ In our subgroup analysis involving children of age groups 1–5 years and 6–17 years, we observed that the GMFR was higher after the first dose as compared to the second dose in children of age group 6–17 years for all three serotypes in both Euvichol-Plus and Shanchol groups. A similar trend was also observed in children of age group 1–5 years for O1 Inaba and Ogawa in both Euvichol-Plus and Shanchol groups. However, for O139, it was only observed in the Shanchol group.

The GMFR and the seroconversion rates of Euvichol-Plus and Shanchol in the current study were lower than the previous clinical trials, probably because the previous studies were conducted in regions with low cholera endemicity.^19^^,^^24^^,^^25^ It has been proven that OCVs are less immunogenic when administered to children or adults living in LMICs, especially cholera-endemic regions, as compared to developed countries.^26^ This includes CVD103-HgR (live-attenuated OCV strain) as well as Shanchol, both of which were found to be less immunogenic in populations living in cholera-endemic regions of India as compared to individuals residing in less endemic settings.^26^^,^^27^ It has been postulated that this could be due to poor sanitation and previous exposure to cholera, resulting in high baseline of serum vibriocidal antibodies, and therefore, the serum titres were not boosted by vaccination. Poor sanitation can also result in small bowel bacterial overgrowth or heavy infection with intestinal helminths, both of which have been shown to affect the immune response to OCVs.^26^ The safety profile of Euvichol-Plus and Shanchol in our study was consistent with the previously published studies.^19^^,^^23^

A matched case–control study was conducted by Sialubanje and colleagues to evaluate the effectiveness of two-dose regimen of Euvichol-Plus administered during a mass vaccination campaign in response to cholera outbreak in Lusaka, Zambia, during 2017–2018.^14^ The study, involving 79 cases and 316 controls, showed that two doses of Euvichol-Plus conferred effective protection and can serve as an intervention in controlling cholera outbreaks.^14^ The adjusted odds ratio (AOR) vaccine effectiveness for two doses of Euvichol-Plus was 81%. Secondary analysis showed that vaccine effectiveness for any dose (one or more dose) was 74%.^14^ Based on the above findings, results from our study, and other published studies (evaluating the effectiveness of Shanchol in outbreak settings and non-inferiority of Euvichol to Shanchol), we believe that Euvichol-Plus can be deployed to effectively control cholera outbreaks in an endemic country like India.^14^^,^^16^^,^^19^ However, larger cohort studies are warranted to demonstrate the real situation effectiveness of Euvichol-Plus in controlling cholera outbreaks.

The WHO position paper in 2017 has recommended the use of OCVs in areas with endemic cholera, in humanitarian crisis with high risk of cholera, and during cholera outbreaks.^3^ On October 19, 2022, the WHO announced that countries having cholera outbreaks will have to administer only a single dose of vaccine instead of the recommended two doses because a high global demand is exhausting international stockpiles.^28^ An effective cholera vaccine has long been the vision of controlling cholera in India.^12^ Moreover, India is not supported by Gavi, the Vaccine Alliance, for OCV.^29^ Current OCV production capacity, particularly of Shanchol, which has been licensed in India, is inadequate considering the population of India and its realistic demand.^6^^,^^12^ As considerable quantity of vaccines manufactured is pre-committed to the WHO global OCV stockpile, the surplus availability for other use is limited.^12^ Also, Sanofi Pasteur has stated that it will discontinue Shanchol production in 2023.^30^ On the other hand, EuBiologics has a larger supply capacity, and its product, Euvichol-Plus, is yet to be licensed in India.^12^ With the expected approval of Euvichol-Plus in India based on this study, it will be possible to address the increasing demand of OCV in India. The lack of a national cholera control plan in India is another major challenge that needs to be addressed as well.^6^

Our study has several strengths. This was the first study of its kind comparing the immunogenicity and safety of Euvichol-Plus with Shanchol in a cholera-endemic country like India. To ensure generalisability, we conducted the study with participants from diverse geographical locations across India. The present study has certain limitations. First, the study was not blinded. This was due to the glass vial presentation of Shanchol and the plastic tube presentation of Euvichol-Plus, which would have been difficult to mask during the study. Second, the study was conducted in a small cohort to seek the market licensure of Euvichol-Plus, which has already been shown to be safe and effective in other countries. Hence, there exist a possibility that a rare adverse event might have been missed out. Third, for the purpose of having a low baseline and to prevent confounding data, we excluded participants with a history of cholera or who already had cholera vaccination, which in entirety is not reflective of real-world data.

In conclusion, Euvichol-Plus appears to be non-inferior to Shanchol in terms of immunogenicity and safety in healthy Indian adults and children. This phase 3 registration study will pave the way for Euvichol-Plus in India and other countries globally where cholera poses a significant public health threat. The plastic tube presentation offers an array of advantages and can be recommended as a suitable alternative to Shanchol, especially given the fact that Sanofi Pasteur has decided to cease its production in 2023.

## Contributors

SS, SSS, BC, SP, SA, YOB, YJL, YP, and KHJ were involved in the literature search as well as in reviewing and editing the manuscript. SD and RKN critically reviewed the manuscript. RKN generated the entire data of bioanalytic testing. SR, CS, VC, CP, NRK, ATC, and AC were the investigators for the clinical trial. AS was involved in the medical monitoring of the clinical trial and safety assessment. AKR conducted the statistical analysis of the clinical data and reviewed the manuscript. NS supervised the clinical operations during the trial. All the authors reviewed, commented on, and approved this manuscript before submission. All the authors had full access to all the data in the trial and had final responsibility for the decision to submit the manuscript for publication.

## Data sharing statement

Data sharing requests will be considered from research groups that submit a research proposal. Requests for de-identified data following manuscript publication should be directed to the corresponding author. Proposals will be evaluated internally, and the data will be shared solely based on scientific merit after signing a data sharing agreement.

## Declaration of interests

SD and RKN are affiliated with ICMR-NICED, Kolkata. A memorandum of agreement was signed between Techinvention Lifecare Private Limited and ICMR-NICED. Laboratory analysis was carried under collaborative project between ICMR-NICED, Kolkata, and Techinvention Lifecare Private Limited, Mumbai. Fund provided by Techinvention Lifecare Private Limited was received by ICMR-NICED for carrying out bioanalytic testing at ICMR-NICED. All other authors have no competing interests.

## Appendix A Supplementary data

Supplementary Table S1

Final OCV Protocol
