# Supplementary material for: Comparison of the immunogenicity and safety of Euvichol-Plus with Shanchol in healthy Indian adults and children: an open-label, randomised, multicentre, non-inferiority, parallel-group, phase 3 trial
Source: Lancet Reg Health Southeast Asia. 2023 Aug 24;19:100256. doi: 10.1016/j.lansea.2023.100256 (PMC10709676; doi:10.1016/j.lansea.2023.100256)
Supplement: Supplementary Table S1 [file mmc1.docx]

**Supplementary Table 1: Demographic characteristics of the study participants**

| **Parameters** | **Categories** | **Euvichol-Plus**  **(N=208)** | **Shanchol**  **(N=208)** | **Total (N=416)** |
| --- | --- | --- | --- | --- |
| Age (Years) | Standard Error | 1·1 | 1·07 | 0·77 |
|  | Median | 17·7 | 18·1 | 17·7 |
|  | Range (Min:Max) | (1:60) | (1:59) | (1:60) |
| Gender | Male | 106 (51%) | 114 (54·8%) | 220 (52·9%) |
|  | Female | 102 (49%) | 94 (45·2%) | 196 (47·1%) |
| Height (cm) | Standard Error | 1·99 | 1·94 | 1·39 |
|  | Median | 154 | 152·7 | 154 |
|  | Range (Min:Max) | (52:184) | (46:182) | (46:184) |
| Weight (kg) | Standard Error | 1·41 | 1·38 | 0·98 |
|  | Median | 50 | 50 | 50 |
|  | Range (Min:Max) | (7:85) | (7·5:90) | (7:90) |
| BMI (kg/m^2^) | Standard Error | 0·32 | 0·35 | 0·24 |
|  | Median | 22·4 | 22·2 | 22·3 |
|  | Range (Min:Max) | (11·73:38·27) | (10·59:42·20) | (10·59:42·20) |
| BMI: Body mass index, N: Number of participants, Min: Minimum, Max: Maximum. Percentages were calculated using the respective column header count as denominator. | | | | |
