# Supplementary material for: Comparison of the immunogenicity and safety of Euvichol-Plus with Shanchol in healthy Indian adults and children: an open-label, randomised, multicentre, non-inferiority, parallel-group, phase 3 trial
Source: Lancet Reg Health Southeast Asia. 2023 Aug 24;19:100256. doi: 10.1016/j.lansea.2023.100256 (PMC10709676; doi:10.1016/j.lansea.2023.100256)
Supplement: Final OCV Protocol [file mmc2.pdf]

CLINICAL STUDY PROTOCOL

A Phase III, open label, multicenter, parallel group, randomized Clinical Study to compare the Immunogenicity and Safety of Euvichol-Plus vaccine with Oral Cholera Vaccine Shanchol in healthy adults and children above age of one year

**Protocol ID:**	**EPV/TLC/P-III/2020**
**Phase of study:**	**Phase III**
**Investigational Drug name:**	**Euvichol- Plus Vaccine**
**Version No.:**	**2.0**
**Version Date:**	**7th March 2021**

**Sponsor :**	**Techinvention lifecare Pvt. Ltd.**<br>1004, The summit business park,<br>Off WEH metro station, Andheri kurla road<br>Andheri East Mumbai 400093. INDIA
**Clinical Research Organization:**	**Clinical Research Network India**<br>B-812, Advant Navis Business Park, Plot #7,<br>Noida-Greater Noida Expressway, Sector 142,<br>Noida, Delhi-NCR, Uttar Pradesh 201305
**Central Lab Details:**	**National Institute of Cholera and Enteric Diseases**<br>P- C.I.T. Scheme XM, Beleghata, 33, CIT Rd,<br>Subhas Sarobar Park, Phool, Kolkata,<br>West Bengal 700010

This document is property of Techinvention Lifecare Pvt Ltd and is confidential. Therefore, it may not be photocopied, either in part or full, or shown to any person not directly associated with the trial, or with the concerned ethics committee, or with the concerned regulatory authority.

Summary of Changes

Topic/ Section	Version 1	Version 2
Study Centers	5-6 centres	8 Centres (as sample size increased)
Study Population – Protocol Synopsis. As per SEC (vaccine) recommendation	300 healthy subjects<br>Cohort 1 subjects ≥ 18 years old<br>Cohort 2 subjects ≥01-<18 years old	416 healthy subjects Cohort 1 subjects ≥ 18 years to 60 years old<br>Cohort 2 subjects ≥ 01- <18 years old
Sample size justification- Protocol Synopsis As per SEC (vaccine) recommendation, that the Non-inferiority margin should be revised to 10% and sample size should be changed accordingly.	If the true seroconversion rate for Euvichol-Plus in study subjects is non inferior to Shanchol with predefined margin of 20%, we will need to study 136 test subjects and control subjects to be able to reject the null hypothesis that the seroconversion rates for Euvichol-Plus and Shanchol subjects are equal with probability (power) 0.9.<br><br>The Type I error probability associated with this test of this null hypothesis is 0.05. we will use an uncorrected chi squared statistic to evaluate this null hypothesis. Anticipating a dropout of 10%, we will plan to enrol 150 subjects in each study arm.	Total Sample size with 10% Dropout is 416. As per published clinical study evaluating immunogenicity of Shancol and Euvichol, the seroconversion rates (4-fold rise in antibody tires) 14 days after first and second dose of vaccine ranges from 73% to 90% in adults and children.<br><br>Assuming seroconversion rate of 70% in the reference arm, power of 90%, alpha of 5% (2-sided) and a non-inferiority margin of 10%, 186 subjects would be required in the test and reference arms to establish non-inferiority of the vaccine.<br><br>Considering a dropout rate of 10%, a total of 416 subjects will be enrolled in the study (208 in Test and 208 in reference arms).<br><br>Approximately 208 subjects enrolled in the study will be adults (≥ 18 to 60 years of age) while remaining 208 would be children (≥1 to <18 years of age).
Inclusion Criteria Second point as per ICMR guidelines on Biomedical research on Children (2017)	1. Individuals aged 1 year to 45 years (both inclusive)<br>2. Subjects aged 11 to 17 years, had to sign an assent for the study and a parent or a legal guardian have to sign the informed consent.	1. Individuals aged ≥ 1 year to 60 years (both inclusive)<br>2. Subjects aged 12 to <18 years, must sign an assent for the study and a parent or a legal guardian have to sign the informed consent. Subject aged 7 to 11 years need to provide an oral consent/assent and the consent form must be signed by parent/legally acceptable representative.Less than 7 years no need of assent, LAR can give consent.
Exclusion Criteria under Protocol summary	Exclusion criteria include 12 criteria while exclusion criteria number 12 was missed out.	Exclusion criteria includes 12 criteria, Inadvertently exclusion criteria number 12 was missed out. Exclusion criteria number 12 included as “If female patient will get pregnant after first vaccination”

Topic/ Section	Version 1	Version 2
6.2<br>As per SEC (vaccine) recommendation	Sample size<br>A total of 300 eligible subjects will be enrolled at visit 1 for the study as per below<br>**Cohort 1 adults:**<br>Test: 75 subjects<br>Reference: 75 subjects<br>**Cohort 2 children:**<br>Test: 75 subjects<br>Reference: 75 subjects<br><br>Shanchol has shown be immunogenic in Indian population in past, with an average seroconversion rate of 68%.<br><br>If the true seroconversion rate for Euvichol-Plus in study subjects non inferior to Shanchol with predefined margin of 20%, we will need to study 136 test subjects and control subjects to be able to reject the null hypothesis that the seroconversion rates for Euvichol-Plus and Shanchol subjects are equal with probability (power) 0.9.<br><br>The Type I error probability associated with this test of this null hypothesis is 0.05. We will use an uncorrected chi squared statistic to evaluate this null hypothesis. Anticipating a dropout of 10%, we will plan to enroll 150 subjects in each study arm.	Sample Size<br>A total of 416 eligible subjects will be enrolled at visit 1 for the study as per below<br>**Cohort 1 adults:**<br>Test: 104 subjects<br>Reference: 104 subjects<br>**Cohort 2 children:**<br>Test: 104 subjects<br>Reference: 104 subjects<br><br>As per published clinical study evaluating immunogenicity of Shancol and Euvichol, the seroconversion rates (4-fold rise in antibody tires) 14 days after first and second dose of vaccine ranges from 73% to 90% in adults and children.<br>Assuming seroconversion rate of 70% in the reference arm, power of 90%, alpha of 5% (2-sided) and a non-inferiority margin of 10%, 186 subjects would be required in the test and reference arms to establish non-inferiority of the vaccine.<br>Considering a dropout rate of 10%, a total of 416 subjects will be enrolled in the study (208 in Test and 208 in reference arms).<br>Approximately 208 subjects enrolled in the study will be adults (≥ 18 to 60 years of age) while remaining 208 would be children (≥1 to <18 years of age).

Topic/ Section	Version 1	Version 2
6.4 Randomization	There will be two study arms, each comprising 150 healthy subjects in both Cohorts 1 and 2	Randomization will be done for Cohort 1 and 2, each comprising of 208 healthy subjects. Each cohort will have two study arms, comprising 104 healthy subjects enrolled into each treatment arm.
6.4 Flow diagram for randomization	page no. 23 of protocol mentioned Cohort 1 as children and cohort 2 as adults.<br>Euvichol-Plus 75 subjects<br>Shanchol 75 subjects	Cohort 1 adults: ≥18 years to 60 years<br>Cohort 2 children: ≥1 to <18 years<br>Euvichol-Plus 104 subjects<br>Shanchol 104 subjects

Investigator List

1. Dr. Sanjay K. Rai
Centre for Community Medicine, AIIMS,
New Delhi-110029

2. Dr. Vinod Chayal
Room no. 405, Dept. of Community Medicine,
Pt. B D Sharma PGIMS, Rohtak, Haryana

3. Dr. Chandramani Singh
Dept. of Community & Family Medicine AIIMS,
Patna Bihar- 801507

4. Dr. Jinen Mukeshbhai Shah
Aartham Multi Super Speciality Hospital, Opp. Polytechnic, NR. Panjarapole cross road, Ambawadi, Ahmedabad. 380006

5. Dr. N. Ravi Kumar
Dept. of Pediatrics, Niloufer Hospital,
Red Hills, Lakdikapool,
Hyderabad-500004, Telangana

6. Dr. Abhishek T. Chavan
Consultant Pediatrician, Jeevan Rekha Hospital,
Dr. B R Ambedkar Road Belagavi-590002

7. Dr. Amit Chawla
Consultant Pediatrician, Prakhar Hospital Pvt Ltd.,
8/219 Arya Nagar, Kanpur, Uttar Pradesh-208002

8. Dr Rambha Pathak
Government Institute of Medical Sciences,
Greater Noida,
Gautam Budh Nagar, UP-201310

TABLE OF CONTENTS

Summary of changes ……………………………………………………………………….2
List of Investigators………………………………………………………………………

1.0 DECLARATION..8
1.1 Investigator Declaration..8
1.2 Sponsor's Declaration..17

2.0 LIST OF ABBREVIATIONS 18

3.0 PROTOCOL SUMMARY 19

4.0 BACKGROUND INFORMATION..24
4.1 Investigational Product Details 26
4.2 Characterization of Study Products 27
4.3 Dosage and Administration 28
4.4 How does it Work?..28
4.5 Adverse Effects...28
4.6 Rationale: 29
4.7 Study Objectives...29
4.8 Study Endpoints: 29

5.0 HANDLING, STORAGE, DISPENSING AND ACCOUNTABILITY PROCEDURES FOR INVESTIGATIONAL PRODUCTS...30
5.1 Investigational Product Receipt and Storage..30
5.2 Dosing & Dispensing 31
5.3 Follow-up 31
5.4 Subjects Compliance 31
5.5 Study Product Accountability 31
5.6 Concomitant vaccines..31
5.7 Concomitant Medication 31

6.0 STUDY DESIGN 32
6.1 Description of Study Design 32
6.2 Sample Size...32
6.3 Blinding 32
6.4 Randomization 32

6.5 Expected Duration of Study: ... 33
6.6 Duration of Protocol therapy ... 33
6.7 Duration of Subject Participation: ... 33

7.0 SELECTION AND WITHDRAWAL OF SUBJECTS ... 35
7.1 Inclusion Criteria ... 35
7.2 Exclusion Criteria: ... 35
7.3 Withdrawal of Subjects ... 36

8.0 STUDY DATA COLLECTION ... 37
8.1 Study Visits: ... 37
8.2 Method of blood collection and storage ... 38
8.3 Central Laboratory ... 38
8.4 Schedule of events ... 38

9.0 PROTOCOL DEVIATIONS AND VIOLATIONS ... 38

10.0 ADVERSE EVENT ... 39
10.1 Adverse Event Monitoring ... 39
10.2 Adverse Event Documentation ... 39
10.3 Severity and Relation of Adverse Event ... 41
10.4 Serious Adverse Events Reporting ... 42
10.5 Follow up of subject with Adverse Events ... 43
10.6 Precautions ... 43

11.0 DATA AND SAFETY MONITORING BOARD ... 43

12.0 STATISTICAL METHODS ... 43

13.0 ETHICAL AND REGULATORY CONSIDERATIONS ... 43
13.1 GCP and Monitoring ... 44
13.2 Ethics Committee Approval ... 44
13.3 Protocol Amendments ... 44
13.4 Written Informed Consent and Subject Information ... 44

14.0 DATA AND SAFETY MONITORING BOARD [DSMB] ... 45

15.0 DATA HANDLING ... 45

16.0 ACCESS TO SOURCE DATA/DOCUMENTS ... 45

17.0 RECORD KEEPING AND ARCHIVING OF DATA ... 45

18.0 STUDY MONITORING 45

19.0 QUALITY CONTROL AND QUALITY ASSURANCE 46

20.0 MAINTENANCE OF LOGS, MONITORING AND AUDIT PLAN 46

19.1 INFORMATION DISCLOSURE AND INVENTIONS 46

19.2 Ownership 46

19.3 Confidentiality 46

21.0 PUBLICATION POLICY 47

22.0 STUDY TERMINATION AND SITE CLOSURE 47

23.0 FINANCING AND INSURANCE 47

24.0 AMENDMENT TO THE PROTOCOL 47

25.0 REFERENCES 49

26.0 ANNEXURE 50

1.0 DECLARATION

1.1 Investigator Declaration

Following are the list of investigators along with signed copy of declaration.

1. Dr. Sanjay K. Rai
Centre for Community Medicine, AIIMS,
New Delhi-110029

2. Dr. Vinod Chayal
Room no. 405, Dept. of Community Medicine,
Pt. B D Sharma PGIMS, Rohtak, Haryana

3. Dr. Chandramani Singh
Dept. of Community & Family Medicine AIIMS,
Patna Bihar- 801507

4. Dr. Jinen Mukeshbhai Shah

Aartham Multi Super Speciality Hospital, Opp. Polytechnic, NR. Panjarapole cross road, Ambawadi, Ahmedabad. 380006

5. Dr. N. Ravi Kumar
Dept. of Pediatrics, Niloufer Hospital,
Red Hills, Lakdikapool,
Hyderabad-500004, Telangana

6. Dr. Abhishek T. Chavan
Consultant Pediatrician, Jeevan Rekha Hospital,
Dr. B R Ambedkar Road Belagavi-590002

7. Dr. Amit Chawla
Consultant Pediatrician, Prakhar Hospital Pvt Ltd.,
8/219 Arya Nagar, Kanpur, Uttar Pradesh-208002

8. Dr Rambha Pathak
Government Institute of Medical Sciences,
Greater Noida,
Gautam Budh Nagar, UP-201310

Protocol # EPV/TLC/P-III/2020,

Techinvention
towards greater health equity

INVESTIGATOR PROTOCOL AGREEMENT

Protocol Title: A Phase III, open label, multicenter, parallel group, randomized Clinical Study to compare the Immunogenicity and Safety of Euvichol-Plus vaccine with Oral Cholera Vaccine Shanchol in healthy adults and children above age of one year.

Protocol Number: EPV/TLC/P-III/2020

By my signature, I confirm that my staff and I have carefully read and understood this protocol or protocol amendment, and agree to comply with the conduct and terms of the study specified herein.

I agree to conduct the study according to this protocol and the obligations and requirements of clinical investigators and all other requirements listed in ICH guidelines. I will not initiate this study without the approval of an Institutional Review Board (IRB) / Independent Ethics Committee (IEC).

I understand that, should the decision be made by the sponsor to terminate prematurely or suspend the study at any time for whatever reason; such decision will be communicated to me in writing. Conversely, should I decide to withdraw from execution of the study, I will communicate immediately such decision in writing to the sponsor.

For protocol amendments, I agree not to implement the amendment without agreement from the sponsor and prior submission to and written approval (where required) from the IRB or IEC, except when necessary to eliminate an immediate hazard to the subjects, or for administrative aspects of the study (where permitted by all applicable regulatory requirements).

[signature] 16-03-2021

Investigator's Signature Date

Investigator's Name डॉ० सजंय के. राय/Dr. Sanjay K. Rai
आचार्य/Professor
सामुदायिक चिकित्सा विज्ञान केन्द्र
Centre for Community Medicine
अ.भा.आ.सं., नई दिल्ली/AIIMS, New Delhi

Address

Protocol # EPV/TLC/P-III/2020,

INVESTIGATOR PROTOCOL AGREEMENT

Protocol Title: A Phase III, open label, multicenter, parallel group, randomized Clinical Study to compare the Immunogenicity and Safety of Euvichol-Plus vaccine with Oral Cholera Vaccine Shanchol in healthy adults and children above age of one year.

Protocol Number: EPV/TLC/P-III/2020

By my signature, I confirm that my staff and I have carefully read and understood this protocol or protocol amendment, and agree to comply with the conduct and terms of the study specified herein.

I agree to conduct the study according to this protocol and the obligations and requirements of clinical investigators and all other requirements listed in ICH guidelines. I will not initiate this study without the approval of an Institutional Review Board (IRB) / Independent Ethics Committee (IEC).

I understand that, should the decision be made by the sponsor to terminate prematurely or suspend the study at any time for whatever reason; such decision will be communicated to me in writing. Conversely, should I decide to withdraw from execution of the study, I will communicate immediately such decision in writing to the sponsor.

For protocol amendments, I agree not to implement the amendment without agreement from the sponsor and prior submission to and written approval (where required) from the IRB or IEC, except when necessary to eliminate an immediate hazard to the subjects, or for administrative aspects of the study (where permitted by all applicable regulatory requirements).

Investigator's Signature: Vinod 11/3/21

Date: 11-03-2021

Investigator's Name: **Dr. Vinod Chayal**

Address: **Room No. 405, Department of Community Medicine, Pt. B.D. Sharma PGIMS, Rohtak, Haryana**

Deptt. of Community Medicine
Pt. B.D. Sharma PGIMS, Rohtak

Protocol # EPV/TLC/P-III/2020,

towards greater health equity

INVESTIGATOR PROTOCOL AGREEMENT

Protocol Title: A Phase III, open label, multicenter, parallel group, randomized Clinical Study to compare the Immunogenicity and Safety of Euvichol-Plus vaccine with Oral Cholera Vaccine Shanchol in healthy adults and children above age of one year.

Protocol Number: EPV/TLC/P-III/2020

By my signature, I confirm that my staff and I have carefully read and understood this protocol or protocol amendment, and agree to comply with the conduct and terms of the study specified herein.

I agree to conduct the study according to this protocol and the obligations and requirements of clinical investigators and all other requirements listed in ICH guidelines. I will not initiate this study without the approval of an Institutional Review Board (IRB) / Independent Ethics Committee (IEC).

I understand that, should the decision be made by the sponsor to terminate prematurely or suspend the study at any time for whatever reason; such decision will be communicated to me in writing. Conversely, should I decide to withdraw from execution of the study, I will communicate immediately such decision in writing to the sponsor.

For protocol amendments, I agree not to implement the amendment without agreement from the sponsor and prior submission to and written approval (where required) from the IRB or IEC, except when necessary to eliminate an immediate hazard to the subjects, or for administrative aspects of the study (where permitted by all applicable regulatory requirements).

[signature] 08-MARCH-2021

Investigator's Signature Date

Investigator's Name Dr. Chandramani Singh, MD

Professor
Department of Community & Family Medicine
AIIMS, PATNA

Address AIIMS. PATNA - 801507

Protocol # EPV/TLC/P-III/2020,

INVESTIGATOR PROTOCOL AGREEMENT

Protocol Title: A Phase III, open label, multicenter, parallel group, randomized Clinical Study to compare the Immunogenicity and Safety of Euvichol-Plus vaccine with Oral Cholera Vaccine Shanchol in healthy adults and children above age of one year.

Protocol Number: EPV/TLC/P-III/2020

By my signature, I confirm that my staff and I have carefully read and understood this protocol or protocol amendment, and agree to comply with the conduct and terms of the study specified herein.

I agree to conduct the study according to this protocol and the obligations and requirements of clinical investigators and all other requirements listed in ICH guidelines. I will not initiate this study without the approval of an Institutional Review Board (IRB) / Independent Ethics Committee (IEC).

I understand that, should the decision be made by the sponsor to terminate prematurely or suspend the study at any time for whatever reason; such decision will be communicated to me in writing. Conversely, should I decide to withdraw from execution of the study, I will communicate immediately such decision in writing to the sponsor.

For protocol amendments, I agree not to implement the amendment without agreement from the sponsor and prior submission to and written approval (where required) from the IRB or IEC, except when necessary to eliminate an immediate hazard to the subjects, or for administrative aspects of the study (where permitted by all applicable regulatory requirements).

[signature] 08/03/2021

Investigator's Signature Date

Prakhar Hospital Pvt. Ltd.
8/219, Arya Nagar,
Kanpur-208002

Investigator's Name Dr. Amit Chawla.

Address Consultant Pediatrician.
Prakhar hospital, KANPUR 208002

Version No 2.0 dated 7th March 2021

Protocol # EPV/TLC/P-III/2020,

INVESTIGATOR PROTOCOL AGREEMENT

Protocol Title: A Phase III, open label, multicenter, parallel group, randomized Clinical Study to compare the Immunogenicity and Safety of Euvichol-Plus vaccine with Oral Cholera Vaccine Shanchol in healthy adults and children above age of one year.

Protocol Number: EPV/TLC/P-III/2020

By my signature, I confirm that my staff and I have carefully read and understood this protocol or protocol amendment, and agree to comply with the conduct and terms of the study specified herein.

I agree to conduct the study according to this protocol and the obligations and requirements of clinical investigators and all other requirements listed in ICH guidelines. I will not initiate this study without the approval of an Institutional Review Board (IRB) / Independent Ethics Committee (IEC).

I understand that, should the decision be made by the sponsor to terminate prematurely or suspend the study at any time for whatever reason; such decision will be communicated to me in writing. Conversely, should I decide to withdraw from execution of the study, I will communicate immediately such decision in writing to the sponsor.

For protocol amendments, I agree not to implement the amendment without agreement from the sponsor and prior submission to and written approval (where required) from the IRB or IEC, except when necessary to eliminate an immediate hazard to the subjects, or for administrative aspects of the study (where permitted by all applicable regulatory requirements).

[illegible] 09-03-2021

Investigator's Signature Date

Investigator's Name Dr. Abhishek Chavan, MD

Address Consultant Peadiatrician.
Jeevan Rekha hospital, Belgaum.

Jeevan Rekha Hospital
Dr. B R Ambedkar Road,
Opp. Civil Hospital
Belagavi-590002

Version No 2.0 dated 7th March 2021

Protocol # EPV/TLC/P-III/2020,

INVESTIGATOR PROTOCOL AGREEMENT

Protocol Title: A Phase III, open label, multicenter, parallel group, randomized Clinical Study to compare the Immunogenicity and Safety of Euvichol-Plus vaccine with Oral Cholera Vaccine Shanchol in healthy adults and children above age of one year.

Protocol Number: EPV/TLC/P-III/2020

By my signature, I confirm that my staff and I have carefully read and understood this protocol or protocol amendment, and agree to comply with the conduct and terms of the study specified herein.

I agree to conduct the study according to this protocol and the obligations and requirements of clinical investigators and all other requirements listed in ICH guidelines. I will not initiate this study without the approval of an Institutional Review Board (IRB) / Independent Ethics Committee (IEC).

I understand that, should the decision be made by the sponsor to terminate prematurely or suspend the study at any time for whatever reason; such decision will be communicated to me in writing. Conversely, should I decide to withdraw from execution of the study, I will communicate immediately such decision in writing to the sponsor.

For protocol amendments, I agree not to implement the amendment without agreement from the sponsor and prior submission to and written approval (where required) from the IRB or IEC, except when necessary to eliminate an immediate hazard to the subjects, or for administrative aspects of the study (where permitted by all applicable regulatory requirements).

______________________________ ______________

Investigator's Signature Date

Investigator's Name

Address

Protocol # EPV/TLC/P-III/2020,

INVESTIGATOR PROTOCOL AGREEMENT

Protocol Title: A Phase III, open label, multicenter, parallel group, randomized Clinical Study to compare the Immunogenicity and Safety of Euvichol-Plus vaccine with Oral Cholera Vaccine Shanchol in healthy adults and children above age of one year.

Protocol Number: EPV/TLC/P-III/2020

By my signature, I confirm that my staff and I have carefully read and understood this protocol or protocol amendment, and agree to comply with the conduct and terms of the study specified herein.

I agree to conduct the study according to this protocol and the obligations and requirements of clinical investigators and all other requirements listed in ICH guidelines. I will not initiate this study without the approval of an Institutional Review Board (IRB) / Independent Ethics Committee (IEC).

I understand that, should the decision be made by the sponsor to terminate prematurely or suspend the study at any time for whatever reason; such decision will be communicated to me in writing. Conversely, should I decide to withdraw from execution of the study, I will communicate immediately such decision in writing to the sponsor.

For protocol amendments, I agree not to implement the amendment without agreement from the sponsor and prior submission to and written approval (where required) from the IRB or IEC, except when necessary to eliminate an immediate hazard to the subjects, or for administrative aspects of the study (where permitted by all applicable regulatory requirements).

Investigator's Signature [signature] Date 8/03/2021

Professor of Paediatric
Department of Paediatric
Niloufer Hospital
Redhills, Lakdikapool
Hyderabad-04 Telangana

Investigator's Name: **Dr. N. Ravi Kumar**

Address
Department of Pediatrics
Niloufer Hospital (Affiliated to Osmania Medical College),
Red Hills, Lakdikapool,
Hyderabad-500004, Telangana.
Mob: +91-9490919293
E-mail ID: ravik1961@yahoo.com

INVESTIGATOR PROTOCOL AGREEMENT

Protocol Title: A Phase III, open label, multicenter, parallel group, randomized Clinical Study to compare the Immunogenicity and Safety of Euvichol-Plus vaccine with Oral Cholera Vaccine Shanchol in healthy adults and children above age of one year.

Protocol Number: EPV/TLC/P-III/2020

By my signature, I confirm that my staff and I have carefully read and understood this protocol or protocol amendment, and agree to comply with the conduct and terms of the study specified herein.

I agree to conduct the study according to this protocol and the obligations and requirements of clinical investigators and all other requirements listed in ICH guidelines. I will not initiate this study without the approval of an Institutional Review Board (IRB) / Independent Ethics Committee (IEC).

I understand that, should the decision be made by the sponsor to terminate prematurely or suspend the study at any time for whatever reason; such decision will be communicated to me in writing. Conversely, should I decide to withdraw from execution of the study, I will communicate immediately such decision in writing to the sponsor.

For protocol amendments, I agree not to implement the amendment without agreement from the sponsor and prior submission to and written approval (where required) from the IRB or IEC, except when necessary to eliminate an immediate hazard to the subjects, or for administrative aspects of the study (where permitted by all applicable regulatory requirements).

Government Institute of Medical Sciences, Greater Noida

Investigator's Signature ____________________ Date 17/03/2021

Investigator's Name Dr. RAMBHA PATHAK

Address Prof. & Head, Dept. of Community Medicine
Govt. Institute of Medical Sciences,
Greater Noida, UP - 201310.

1.2 Sponsor's Declaration

We, on behalf of Sponsor have read, understood & approve this Protocol. We agree to comply with all requirements regarding the obligations of Sponsor and current version of the ICH-Guidelines, New Clinical Trial Regulations March 2019, ICMR guidelines and the principles enunciated in the Declaration of Helsinki and regulations in force in India where the study is being conducted.

We agree to maintain a list of appropriately qualified persons to whom we shall delegate significant trial related duties. We shall ensure that all persons assisting us with the trial are adequately informed about the protocol and any amendments, and their trial related duties and functions.

Mr Syed Ahmed

CEO and Director

TECHINVENTION LIFECARE PVT LTD MUMBAI INDIA

7th March 2021

Sponsor Representative: **Date**

Techinvention Lifecare Pvt. Ltd.
1004, The summit business park,
Off WEH metro station, Andheri kurla road
Andheri east Mumbai 400093. INDIA

2.0 LIST OF ABBREVIATIONS

Abbreviation	:	Full Name
**ADR**	:	Adverse Drug Reaction
**AEFI**	:	Adverse Event Following Immunization
**AE**	:	Adverse Event
**CI**	:	Confidence Interval
**CRA**	:	Clinical Research Associate
**CRF**	:	Case Report Form
**CRO**	:	Clinical Research Organization
**CT**	:	Clinical Trial
**DSMB**	:	Data and Safety Monitoring Board
**DCGI**	:	Drug Controller General India
**EC**	:	Ethics Committee
**EU**	:	ELISA Units
**GCP**	:	Good Clinical Practice
**GMT**	:	Geometric Mean Titer
**GMC**	:	Geometric Mean Concentration
**GMP**	:	Good Manufacturing Practice
**IB**	:	Investigator’s Brochure
**ICF**	:	Informed Consent Form
**ICH**	:	International Council for Harmonisation of Technical Requirements for Pharmaceuticals for Human Use
**ICMR**	:	Indian Council of Medical Research
**IEC**	:	Independent Ethics Committee
**IRB**	:	Institutional Review Board
**IU**	:	International Unit
**LAR**	:	Legally Acceptable Representative
**Mcg**	:	Microgram
**OCV**	:	Oral Cholera Vaccine
**q.s.**	:	Quantum Satis
**QA**	:	Quality Assurance
**SAE**	:	Serious Adverse Event
**SAP**	:	Statistical Analysis Plan
**SD**	:	Standard Deviation
**SOP**	:	Standard Operating Procedure
**TEAE**	:	Treatment Emergent Adverse Events
**WHO**	:	World Health Organization
**WMA**	:	World Medical Association

3.0 PROTOCOL SUMMARY

**Title:**	A Phase III, open label, multicenter, parallel group, randomized Clinical Study to compare the Immunogenicity and Safety of Euvichol-Plus vaccine with Oral Cholera Vaccine Shanchol in healthy adults and children above age of one year.
**Phase of Development:**	A Pivotal Phase III Immunogenicity and Safety study for population bridging
**Indication**	Prevention of Cholera caused by *Vibrio cholerae*
**Background:**	Cholera is a significant public health problem in many developing countries. The WHO recommends immunization with cholera vaccines as the complementary management strategy.<br>This study is related to a bivalent (O1 and O139) inactivated whole cell Oral Cholera Vaccine (OCV) containing killed whole cells of *Vibrio cholerae* O1 and *Vibrio cholerae* O139. A two-dose regimen of this vaccine was shown to be well-tolerated and immunogenic against *Vibrio cholerae*.
**Study Centers:**	8 Centers
**Objectives:**	**Primary:**<br>To evaluate the immunogenicity of Euvichol-Plus in healthy adults and children as compared to comparator Oral Cholera Vaccine Shanchol<br>**Secondary:**<br>To evaluate and compare the safety of Euvichol-Plus with Shanchol in healthy adult and children.
**Investigational Products:**	Test(A): Euvichol-Plus<br>Comparator(B): Shanchol<br>Two doses of vaccine (1.5 ml per dose) either Euvichol-Plus or Shanchol will be given to subject as per randomization at an interval of two weeks, administered orally.
**Study Design**	Open label, multi-centric, randomized, parallel group and comparative study.
**Study Population**	416 healthy subjects in step wise two cohorts will be enrolled for the study:<br>✓ Cohort 1 subjects ≥ 18 years to 60 years old<br>✓ Cohort 2 subjects ≥ 01 to <18 years old

	DSMB will review the 14 days post vaccination safety data of the subjects of cohort 1 and accordingly give its recommendations for the enrolment of cohort 2.
**Sample Size Justification**	Total Sample size with 10% Dropout is 416.<br>As per published clinical study evaluating immunogenicity of Shancol and Euvichol, the seroconversion rates (4-fold rise in antibody tires) 14 days after first and second dose of vaccine ranges from 73% to 90% in adults and children.<br>Assuming seroconversion rate of 70% in the reference arm, power of 90%, alpha of 5% (2-sided) and a non-inferiority margin of 10%, 186 subjects would be required in the test and reference arms to establish non-inferiority of the vaccine.<br>Considering a dropout rate of 10%, a total of 416 subjects will be enrolled in the study (208 in Test and 208 in reference arms).<br>Approximately 208 subjects enrolled in the study will be adults (≥ 18 to 60 years of age) while remaining 208 would be children (≥1 to <18 years of age).
**Treatment Duration**	28 days
**Study Methodology**	Number of doses and intervals: two doses/Day 0 and Day 14<br>- Method of administration: oral administration<br>- Dose of vaccine to be administered: 1.5 mL/dose<br>- Observational period: 4 weeks (up to 2 weeks after the 2nd dose)<br>- Number of visits: 5 visits<br>(1) Visit 1: screening and 1st dosing (Day 1)<br>(2) Follow up 1 (Telephonic visit – till 3 days after the 1st dose)<br>(3) Visit 2: 2nd dosing (14 + 3 days)<br>(4) Follow up 2 (Telephonic visit – till 3 days after the 2nd dose)<br>(5) Visit 3: 2 weeks after the 2nd dosing (28 + 3 days)<br>This will be carried out in healthy adults and children. Randomization will be stratified according to age ≥18 years to 60 years, ≥1 to <18 years.<br>According to the pre-generated randomization table, the subjects will be randomized to the test and comparator groups (Visit 1) and will be given either the test vaccine or the comparator vaccine. For immunogenicity assessment, the blood sample will be taken at Visit 1 (prior to investigational product dosing), Visit 2 (prior to investigational product dosing), and Visit 3 (after two weeks of the second dose).

	At both follow up telephonic visits after vaccination, the appointed field officer will contact subjects via telephone for 3 consecutive days after the dosing of the vaccine. For safety assessment, the subjects and/or parents/legally acceptable representatives will be instructed to record on the subject diaries adverse events that occur after investigational product dosing.
**Inclusion Criteria**	1. Individuals aged 1 year to 60 years both inclusive<br>2. Voluntary written informed consent form for study participation provided by an individual or his/her legally acceptable representative. Subjects aged 12 to <18 years, must sign an assent for the study and a parent or a legal guardian have to sign the informed consent. Subject aged 7 to 11 years need to provide an oral consent/assent and the consent form must be signed by parent/legally acceptable representative. Less than 7 years no need of assent, LAR can give consent<br>3. An individual who can be followed up during the study period and is capable of complying with the study requirements.<br>4. Healthy subjects as determined by: Medical history, Physical examination and Clinical judgment of the investigator
**Exclusion Criteria**	**Any one of the following**<br>1. History of cholera vaccinations or history of cholera.<br>2. History of hypersensitivity reactions to other preventative vaccinations.<br>3. Immune function disorders including immunodeficiency diseases.<br>4. 38℃ or higher body temperature measured prior to investigational product dosing.<br>5. Diarrhea or abdominal pain lasting 2 weeks or longer within 6 months prior to study initiation. Also, administration of antidiarrheal drugs or antibiotics to treat diarrhea within 1 week prior to study initiation.<br>6. Abdominal pain, nausea, vomiting, or decreased appetite within 24 hours prior to study initiation.<br>7. Pregnant or lactating women should be excluded by performing urine pregnancy test during screening.<br>8. Children vaccinated within 1 month prior to study initiation or planned vaccination during the study.<br>9. Participants in another clinical trial with investigational product dosing within 6 months prior to study initiation.<br>10. An individual thought to have difficulty in participating the study due to severe chronic diseases, based on the judgment of the investigator.

	11. An individual thought to have difficulty in participating the study due to other reasons, based on the judgment of the investigator<br>12. If female patient will get pregnant after first vaccination.
**Endpoints**	**Primary immunogenicity endpoint:**<br>Assessment of seroconversion rate<br>• The proportion of subjects with at least 4 times higher anti-*V. cholerae* O1 Ogawa and Inaba antibody titer at 2 weeks after the second dose (Visit 2) as compared to baseline titers prior to investigational product dosing (Visit 1).<br>**Secondary immunogenicity endpoints:**<br>• The proportion of subjects with at least 4 times higher anti-*V. Cholerae* O139 antibody titer at 2 weeks after the second dose (Visit 2) as compared to baseline titers prior to investigational product dosing (Visit 1).<br>• Geometric Mean Titer (GMT) and Geometric Mean Ratio (GMR) as measured by anti-*V. Cholerae* O1 Ogawa and Inaba antibody titer at 2 weeks after the second dose (Visit 2) as compared to baseline titers prior to investigational product dosing (Visit 1).<br>• Geometric Mean Titer (GMT) and Geometric Mean Ratio (GMR) as measured by anti-*V. Cholerae* O139 antibody titer at 2 weeks after the second dose (Visit 2) as compared to baseline titers prior to investigational product dosing (Visit 1).<br>**Safety endpoints:**<br>From enrolment until end of the study period in:<br>• Solicited adverse events (Day 0 ~ 3) post each vaccination dose and Unsolicited adverse events including abnormal vital signs and physical examination, throughout the study.<br>• SAE including abnormal vital signs and physical examination, throughout the study.
**Statistical Analysis**	Data obtained from the study subjects will be analyzed in three forms; safety set, Intention to Treat (ITT) set, and Per Protocol (PP) set. Descriptive statistics will be used to define baseline characteristics, Mean and SD will be used to report AEs<br>Seroconversion rates will be compared using the chi-square test with Yates correction or the Fisher's exact test<br>Serum vibriocidal titers will be reported using Geometric mean titers (GMT) and Geometric mean fold rise (GMFR). A p-value of less than 0.05 will be considered the threshold to claim statistical significance.

Table 1 Schedule of Study Procedures and Assessments

	**Visit 1** Screening & Treatment initiation Week 1 (Day 01) Baseline/Enrollment	**Telephonic follow up 1** (Day 0-3 from the first dose)	**Visit 2** Second dose Week 2 (Day 14 + 3 days)	**Telephonic follow up 2** (Day 0-3 from the second dose)	**Visit 3** End Of Study Week 4 (Day 28+3 days)
Informed Consent	✓				
Inclusion / Exclusion Criteria	✓	✓			
Demographic data	✓				
Medical History	✓				
Medication history	✓				
Physical and systemic Examination	✓		✓		✓
Vital Signs[1](Pulse, RR and BP) & Temp.	✓		✓		✓
Anti Cholera antibodies	✓		✓		✓
IP Dosing	✓		✓		
Adverse Events	✓	✓	✓	✓	✓
Drug accountability	✓		✓		
Concomitant Medication	✓	✓	✓	✓	✓
Diary card dispensing	✓		✓		
Diary Card Retrieve			✓		✓
End of study					✓

[1] Vital signs, including pulse rate, supine blood pressure (except screening visit when sitting BP will be measured) and oral temperature, will be performed at all visits. Blood pressure and pulse rate will be measured after the subject has been lying supine for at least 5 minutes.

4.0 BACKGROUND INFORMATION

Globally an estimated 3 million cases and 95,000 deaths occur yearly due to cholera. Cholera has been endemic in the deltas of Ganges and Brahmaputra of eastern India and Bangladesh for most part of recorded history[1.] The causative organism Vibrio cholerae O1 has been responsible for large epidemics and even pandemics[2]. Environmental and climate variables have also linked to cholera epidemics with biannual peaks[3]. Several studies of Oral Cholera Vaccine (OCV) have been have conducted to ascertain its safety, efficacy, effectiveness, field feasibility and acceptance in high-risk urban populations. In our neighboring country, experts opine that approximately 66 million people are at risk of cholera in Bangladesh with an estimated 4500 deaths annually. In India, from published sources, it was observed that there were 222,038 cases of reported cholera during a 10-year period (1997–2006). This figure is about six times higher than the number reported to WHO (37,783) over the same period. According to data from population-based diarrhea surveillance in an endemic area of Kolkata, the incidence of cholera was 2.2 cases per 1000 people per year[5].

While long-term intervention to improve water and sanitation should be the mainstay of cholera-control measures, the use of oral cholera vaccines is recommended by WHO to obtain short-term effect for an immediate response[6].International Vaccine Institute (IVI) in Seoul reformulated existing Oral Cholera Vaccine (OCV) which was tested in clinical trials in both Vietnam and India and was demonstrated to be safe [11] and more immunogenic compared of ORC-Vax [12] of VaBiotech Vietnam.

To ensure the reformulated vaccine could be made available internationally, IVI facilitated a technology transfer between VaBiotech and Shantha Biotechnics Ltd., a private biotech company in Hyderabad, India (acquired by Sanofi in 2009), a country with a national regulatory authority certified as fully functional by WHO. In parallel with the technology transfer, IVI, in collaboration with India’s National Institute of Cholera and Enteric Diseases (NICED), conducted a cluster-randomized, placebo-controlled efficacy trial with the reformulated vaccine in Kolkata, India. The two-dose vaccine was shown to provide a protective efficacy of 65% for at least 5 years[7] and this was the first ever demonstration of strong sustained protection by an oral cholera vaccine. In 2009, the vaccine was licensed in both Vietnam (mORCVAX™) and India (Shanchol), and in 2011, Shanchol was approved by the WHO Prequalification. Several clinical trials have been done on Shanchol vaccine in India in past as summarized below;

- **Immunogenicity study of OCV, Kolkata, India (NCT00419133**):

An open label randomized, placebo-controlled trial was conducted to assess the immune responses following one and two doses of the bivalent killed, whole-cell, oral cholera vaccine among adults and children in Kolkata, India. The study assessed whether receipt of a single dose of OCV is as immunogenic as receipt of two doses among adults and children residing in cholera-endemic areas in Kolkata. In addition, the safety of the OCV was also assessed after each dose. The rates of seroconversion after the second dose were found to be 46% of adults and 82% of children.[8]

- **Study on dose-flexibility of OCV, Kolkata, India (NCT 01233362):**[11]

  The objective of this trial was to compare safety and serum vibriocidal antibody responses in participants receiving two OCV doses either 14 days or 28 days apart. It was a double-blind, RCT conducted at the Clinical Trials Unit of the National Institute of Cholera and Enteric Diseases (NICED). Participants were stratified by age group (1–5y, 6–10y, 11–17y, and ≥18y). A total of 356 participants (178 children, 178 adults) were recruited. No statistically significant difference was noted between intervention arms in seroconversion or geometric fold rise. The GMF rise from baseline was higher for O1 Inaba in adults, after receipt of the first dose (6.8 and 8.9 respectively in the 14- and 28-day interval arms) compared to receipt of the second dose (4.6 and 4.7 respectively). In children, the responses were more pronounced with GMF rise from baseline after first dose in both the arms being 29.7 and 20.8 respectively. The GMF rise after second dose was 17.5 and 10.7 respectively

- **Non-inferiority trial on vibriocidal response, Kolkata, India (NCT01579448):**[19]

  A nested, open-label controlled trial of OCV was conducted among healthy non-pregnant subjects aged 6–14 years and >15 years who were initially immunized with two doses of vaccine or placebo five years before as part of a large randomized controlled trial of an oral cholera vaccine. All participants who were in the placebo arm of the phase III efficacy trial were scheduled to receive two doses of vaccine at the end of the trial period. Since unbinding was performed to conduct analysis and identify all individuals who needed to be given the vaccine, an open label trial design was employed in this study. Seroconversion rates were 66% and 69% following two-dose regimens in the boosting and primary immunization arms (boosting arm was non inferior to primary immunization arm). Seroconversion rate was 71% following one-dose regimen in boosting arm and was non inferior to two-dose regimens in the primary immunization arm. When comparing the immune responses between young children (6–14 years) with older children and adults (>15 years), there was no significant difference in the geometric mean fold rise or the percentage who seroconverted in each intervention arm following two doses of OCV.

- **Safety and Immunogenicity of OCV, Vellore, India (NCT00760825)**

  This is open-label study to assessed the safety and immunogenicity of two doses (14 days apart) of an indigenously manufactured, killed, bivalent (Vibrio cholerae O1 and O139), whole-cell oral cholera vaccine in healthy adults (n = 100) and children (n = 100) in a cholera endemic area (Vellore, South India) to fulfill post-licensure regulatory requirements and post-World Health Organization (WHO) prequalification commitments. This study was an open-label, post-licensure, mono-center trial in healthy adults aged 18–40 years and children aged 1–17 years. No serious adverse events were reported during the study. Commonly reported solicited adverse events were headache and general ill feeling. Seroconversion rates after the first and second dose in adults were 67.7% and 55.2%, respectively, against O1 Inaba; 47.9% and 45.8% against O1 Ogawa; and 19.8% and 20.8% against O139. In children, seroconversion rates after the first and second dose were 80.2% and 68.8%, respectively, against O1 Inaba; 72.9% and 67.7% against O1 Ogawa; and 26.0% and 18.8% against O139. The geometric mean titers against O1 Inaba, O1 Ogawa, and O139 in both adults and children were significantly higher after each vaccine dose compared to baseline titers ($P < 0.001$; for both age groups after each dose versus baseline). The seroconversion rates for O1 Inaba, O1 Ogawa, and O139 in both age groups were similar to those in previous studies with the vaccine.

In conclusion, the killed, bivalent, whole-cell oral cholera vaccine has a good safety and reactogenicity profile, and is immunogenic in healthy adults and children.[16]

In addition, IVI reached out to several Developing Countries Vaccine Manufacturers (DCVMs) for technology transfer and product development partnerships, and transferred OCV technology to EuBiologics Co., Ltd in South Korea[20]. After the lab scale technology transfer IVI and EuBiologics initiated the research and development for fed batch production and in February 2011, EuBiologics successfully scaled up the process to manufacturing scale (30 L fermentation) and in December the same year to 100 L fermentation.

Preclinical toxicity studies were conducted in early 2011, and by September 2012, a phase I study was initiated by EuBiologics in Korea. In February 2013, the study was completed, confirming that their killed whole-cell OCV was safe, well-tolerated, and immunogenic[21].

The phase I results enabled EuBiologics to proceed with a randomized controlled phase III trial to assess the safety and immunogenicity of their vaccine as compared to Shanchol This trial was conducted in the Philippines and immunogenicity testing was performed at the IVI labs in Seoul. The clinical study was completed in August 2014, and demonstrated that two doses of the EuBiologics' vaccine induced vibriocidal responses comparable to those elicited by Shanchol[22]. Based on these results, an export-only licensure application was submitted to Korea's Ministry of Food and Drug Safety (MFDS) in September 2014, and in January 2015 the vaccine (Euvichol-Plus) was approved.

In late January 2015, EuBiologics, submitted a dossier for WHO prequalification. By the end of the year, Euvichol-Plus was WHO-prequalified, making it the second vaccine from the IVI pipeline to be WHO-prequalified. This formulation of Euvichol-Plus was produced in a 100-liter fermenter, giving a production capacity of approximately 6 million doses per year. The company continued to make improvements to the vaccine. In 2014, EuBiologics increased production capacity by investing in a 600-liter fermenter and scaling up to enable production of up to 25 million doses per year by 2018. It also slightly changed the formulation by removing thimerosal, which was considered unnecessary in a single dose oral vaccine. This new variation of Euvichol-Plus obtained WHO prequalification in September 2016.

To further improve Euvichol-Plus, EuBiologics changed the presentation of the vaccine from conventional glass vials to plastic tubes (Euvichol-Plus, also thimerosal free), to facilitate delivery in emergency situations or humanitarian campaigns. The new plastic packaging of Euvichol-Plus reduces the vial's volume by nearly 30 percent and weight by over 50 percent, allowing easier transport and distribution of the vaccine and waste management. Compared with glass vials, the plastic packaging is also easier to open and administer.

4.1 Investigational Product Details

Test vaccine (A): Euvichol-Plus of EuBiologics Co., Ltd., BioVenture Plaza 4, 56 Soyanggang-ro, Chuncheon-si, Gangwon-do, South Korea

Comparator vaccine (B): Shanchol of Sanofi Pasteur India Private Limited EI-223, T.T.C. Industrial Area, Mahape, Navi Mumbai.

4.2 Characterization of Study Products

Each dose of Euvichol-Plus oral dose of 1.5 mL contains

Function	Ingredients	Amount	Reference
Active ingredients	*V.cholerae* O1 Inaba Cairo 48 classical biotype, Heat inactivated	300 L.E.U*	In-house
	V.cholerae O1 Inaba Phil 6973 El Tor biotype, Formalin inactivated	600 L.E.U	In-house
	V.cholerae O1 Ogawa Cairo 50 classical biotype, Formalin inactivated	300 L.E.U	In-house
	V.cholerae O1 Ogawa Cairo 50 classical biotype, Heat inactivated	300 L.E.U	In-house
	V.cholerae O139 4260B, Formalin inactivated	600 L.E.U	In-house
Excipients	Sodium phosphate dibasic dehydrate	4.68 mg	Ph. Eur.
	Sodium phosphate monobasic dehydrate	0.97 mg	Ph. Eur.
	Sodium chloride	12.75 mg	Ph. Eur.
	Water for injection	q.s. to 1.5 mL	Ph. Eur.

*L.E.U: Lipopolysaccharide ELISA Units

Each dose of Shanchol oral dose of 1.5 ml contains

Active ingredients	Quantity
V. cholerae O1 Inaba E1 Tor strain Phil 6973 formaldehyde killed	600 EU of LPS#
V.cholerae O1 Ogawa classical strain Cairo 50 heat killed	300 EU of LPS
V.cholerae O1 Ogawa classical strain Cairo 50 formaldehyde killed	300 EU of LPS
V.cholerae O1 Inaba classical strain Cairo 48 heat killed	300 EU of LPS
V.cholerae O139 strain 4260B formaldehyde killed	600 EU LPS
**Excipients**	
Thiomersal I.P.	Not more than 0.02% (w/v)
Buffer	q.s. to 1.5 mL

Elisa Units (EU) of lipopolysaccharide (LPS)

4.3 Dosage and Administration

- Number of doses and intervals: two doses/Day 0 and Day 14
- Method of administration: oral administration
- Dose of vaccine to be administered: 1.5 mL/dose

4.4 How does it Work?

It has been shown to be effective to administer the vaccine orally, which induces local immunity. The vaccine acts locally in the gastrointestinal tract to induce an IgA antibody response (including memory) comparable to that induced by cholera disease itself. The antibacterial intestinal antibodies prevent the bacteria from attaching to the intestinal wall thereby impeding colonization of *Vibrio cholerae* O1 and *Vibrio cholerae* O139. The protection against cholera is specific for both biotype and serotype

4.5 Adverse Effects

The following adverse events are known to occur with Shanchol use. Acute Gastroenteritis, Diarrhea, Fever, Vomiting, Abdominal pain, Itching, Rash, Nausea, Weakness, Cough, Vertigo, Dryness of mouth, Oral ulcer (rare), Sore throat (rare) and Yellowing of urine (rare). It has been observed that the incidence of adverse events is less after the second dose as compared to the first Special Warnings and Precautions.

The following adverse events are known to occur with Euvichol-Plus. Where, 2,999 healthy children and adults (1-40 years) were participated in the clinical study for evaluating the safety.

1. After taking the vaccines, during first 7 days, the most frequently reported adverse drug reactions in the clinical trial were headache, fever, diarrhea, Nausea/Vomiting and Myalgia and 102 subjects (3.40%) among 2,999 subjects were reported.
2. After taking the vaccines, adverse drug reactions were examined for a period of 28 days. 69 subjects (2.30%) among 2,999 subjects were reported with the adverse effects, and Gastrointestinal disorders were reported the highest numbers i.e., 35 subjects (1.17%). The adverse drug reactions during the study (28 days) were as follows,

System Organ Class	Uncommon	Rare
Gastrointestinal disorders	Abdominal pain, Toothache Diarrhea	Vomiting, Upper abdominal pain
General disorders and administration site condition	Pyrexia	Thirst
Infection and infestations	Nasopharyngitis	Gastroenteritis
Nervous system disorders	Headache	Dizziness
Respiratory, thoracic and meditational disorders	Cough	Oropharyngeal pain
Skin and subcutaneous tissue disorders	Pruritus	Rash macular
Musculoskeletal and connective tissue disorders	-	Arthralgia, Neck pain, Pain in extremity
Vascular disorders	-	Flushing

3. Serious adverse event did not occur during the clinical trial period.

4.6 Rationale:

Our intention is to seek licensure of Euvichol-Plus in India, which is being manufactured under PIC(S) GMP in Korea by EuBiologics. Vaccine has been registered in multiple countries, with WHO Pre-qualification for use in subjects 1 year and above. Euvichol-Plus has been procured from UN agencies, comprising more than 70% of OCV supply to cholera endemic countries worldwide. Proposed registration study in India will allow use of new Euvichol-Plus in India which is similar in composition to Shanchol but come in a single use plastic tube presentation, for easy administration.

4.7 Study Objectives

Primary:

- To evaluate the immunogenicity of Euvichol-Plus in healthy adults and children as compared to WHO prequalified comparator Oral Cholera Vaccine Shanchol

Secondary:

- To evaluate and compare the safety of Euvichol-Plus with Shanchol in healthy adults and children.

4.8 Study Endpoints:

Primary immunogenicity endpoint:

Assessment of seroconversion rate

- The proportion of subjects with at least 4 times higher anti-*V. cholerae* O1 Ogawa and Inaba antibody titer at 2 Weeks after the second dose (Visit 2) as compared to baseline titers prior to investigational product dosing (Visit 1).

Secondary immunogenicity endpoints

- The proportion of subjects with at least 4 times higher anti-*V. Cholerae* O139 antibody titer at 2 Weeks after the second dose (Visit 2) as compared to baseline titers prior to investigational product dosing (Visit 1).

- Geometric Mean Titer (GMT) and Geometric Mean Ratio (GMR) as measured by anti-*V. Cholerae* O1 Ogawa and Inaba antibody titer at 2 Weeks after the second dose (Visit 3) as compared to baseline titers prior to investigational product dosing (Visit 1).
- Geometric Mean Titer (GMT) and Geometric Mean Ratio (GMR) as measured by anti-*V. Cholerae* O139 antibody titer at 2 Weeks after the second dose (Visit 3) as compared to baseline titers prior to investigational product dosing (Visit 1).

Safety endpoints:

- Solicited adverse events (Day 0 ~ 3) and Unsolicited adverse events (Day 0 ~ 28) including abnormal vital signs and physical examination.
- SAE (Day 0 ~ 28) including abnormal vital signs and physical examination.

5.0 HANDLING, STORAGE, DISPENSING AND ACCOUNTABILITY PROCEDURES FOR INVESTIGATIONAL PRODUCTS

5.1 Investigational Product Receipt and Storage

Sponsor should supply sufficient quantity of investigational products, for administration to subjects as well as retention purpose. The investigational products should be supplied in sealed packages with appropriate label and certificates of analysis. These investigational products will be kept under controlled access at CRO

The investigational vaccines will be stored under controlled conditions at 2-8°C.

The clinical study material for the study must be used in accordance with the protocol.

The following information will be displayed on dispensing/dosing label prepared for this study (Not limited to):

- Randomization number
- Visit number
- Route of administration
- Storage details
- Batch Number
- Manufacturing date
- Expiry date
- For clinical trial purpose only

Sponsor's Name & Address:

Techinvention lifecare Pvt. Ltd.,

1004, The summit business park,

Off WEH metro station, Andheri kurla road

Andheri east Mumbai 400093. INDIA

5.2 Dosing & Dispensing

1.5 ml/dose which will be administered on Day 0 and Day 14.

5.3 Follow-up

Follow-up is planned for a period of 2 weeks after each vaccination.

5.4 Subjects Compliance

Window period of +3 days will be allowed for visit 2, and visit 3. Subjects not reporting to the hospital or reporting late by more than 3 days for second dose of vaccination or post vaccination follow-up visit after second dose will be excluded from immunogenicity analysis. All the subjects receiving at least 1 dose will be included in safety analysis.

5.5 Study Product Accountability

The principal investigator shall keep and maintain complete and accurate records of investigational material. Records showing the receipt and disposition of all materials shall include an IP accountability log and vaccine administration log. An IP accountability log shall include date on which shipment was received, batch no., manufacturing and expiry date. Vaccine administration log shall include enrollment ID, product arm, date and time of vaccination and identification of vaccine administrator. All unused investigational medication will be returned to the Sponsor with proper records. Applicable SOPs as per sponsor norms shall be followed thereafter.

5.6 Concomitant vaccines

No concomitant vaccination will be allowed during the study period.

5.7 Concomitant Medication

All concomitant medication, including non-study medicines, being taken by the subjects at enrolment and during this clinical trial are to be regarded as concomitant medications and must be documented on the "Concomitant Medications Record" section of CRF.

Antipyretic medication (Paracetamol) may be administered in case of fever, but prophylactic use of antipyretic medication is not permitted. In case if any other medication is administered to the subject during the course of the study, the decision to continue or discontinue the subject in the study would be taken by the investigator in consultation with sponsor. The decision would be based on whether the administered medication is known to interfere with the immune response.

The following concomitant treatments are discouraged and, if used, will lead to subject's withdrawal during this study (see exclusion criteria as well):

1. Systemic steroids
2. Other immune-suppressive agents
3. Blood or plasma derivatives including immunoglobulin.

6.0 STUDY DESIGN

6.1 Description of Study Design

Open Label, multi-centric, randomized, parallel group, comparative study. The purpose of this study is to assess and compare the Immunogenicity and safety of Euvichol-Plus with Shanchol vaccine in aged 1 and above, in Indian population.

DSMB

There will be a DSMB meeting which will analyze the 14 days post vaccination safety data of the subjects of the cohort 1 accordingly give its recommendations for the enrollment of cohort DSMB will also review the 14 days post vaccination safety data of the subjects of cohort. Subsequent, DSMB meeting will take place as required.

6.2 Sample Size

A total of 416 eligible subjects will be enrolled at visit 1 for the study as per below

Cohort 1 adults:

Test: 104 subjects

Reference: 104 subjects

Cohort 2 children:

Test: 104 subjects

Reference: 104 subjects

Total Sample size with 10% Dropout is 416. As per published clinical study evaluating immunogenicity of Shancol and Euvichol, the seroconversion rates (4-fold rise in antibody tires) 14 days after first and second dose of vaccine ranges from 73% to 90% in adults and children. Assuming seroconversion rate of 70% in the reference arm, power of 90%, alpha of 5% (2-sided) and a non-inferiority margin of 10%, 186 subjects would be required in the test and reference arms to establish non-inferiority of the vaccine.

Considering a dropout rate of 10%, a total of 416 subjects will be enrolled in the study (208 in Test and 208 in reference arms).

Approximately 208 subjects enrolled in the study will be adults (≥ 18 to 60 years of age) while remaining 208 would be children (≥1 to <18 years of age).

6.3 Blinding

The study is planned as open label study. Trial subjects and investigator will be aware of treatments received as whether subject is taking Euvichol-Plus or Shanchol.

6.4 Randomization

Randomization will be done in the Cohort 1 and 2, each comprising of 208 healthy subjects. Each cohort will have two study arms, comprising 104 healthy subjects enrolled into each treatment arm. The enrolled subjects will be randomly allocated in a 1:1 ratio to receive either of the following:

- Euvichol-Plus
- Shanchol

Biostatistician will prepare the randomization list for the study available resources to prevent biasness. After confirmation that the subject meets the eligibility criteria for enrolment in the clinical trial, Subjects would be assigned a randomization number sequentially in the order in which they enter the study by the Investigator.

Figure 1 Flow diagram for randomization

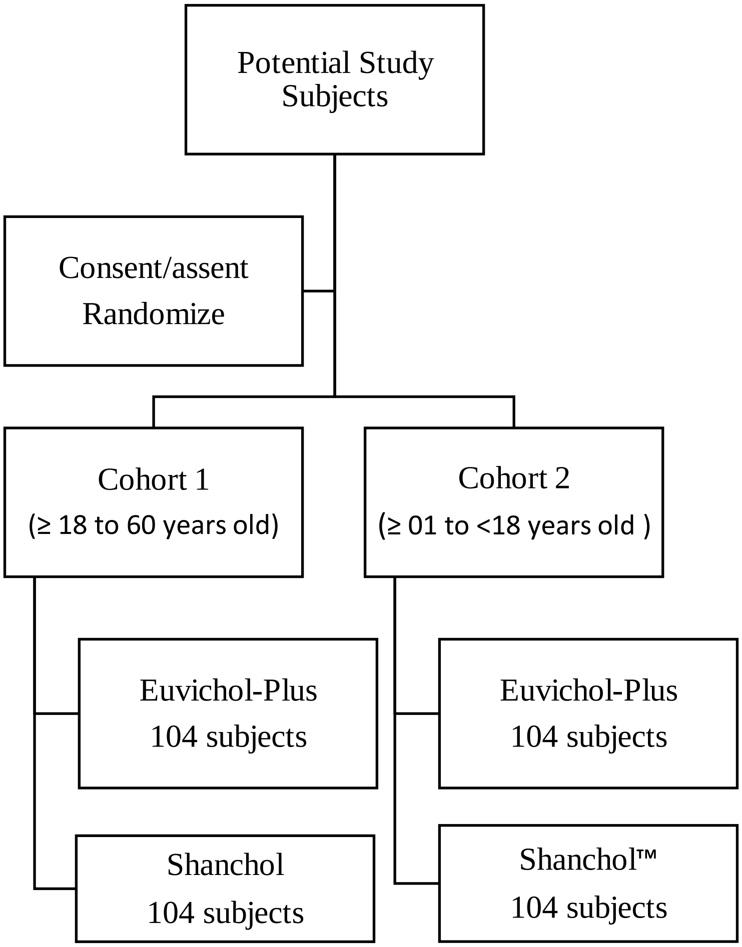

6.5 Expected Duration of Study:

Approximately 7-8 months from the date of study initiation.

6.6 Duration of Protocol therapy

4 weeks (2 vaccinations, each 2 weeks apart with a window period of 3 days at each dose).

6.7 Duration of Subject Participation:

Each subject will have a total of 5 study visits, which are as follows:

- Visit 1: Screening Enrolment and First Vaccination (Day 0)
- Follow Up 1 (Telephonic Visit)
- Visit 2: Second Vaccination Visit (Day 14 + 3)
- Follow Up 2(Telephonic Visit)
- Visit 3: End of Study(EOS)Visit (Day 28 + 3)

STUDY FLOWCHART

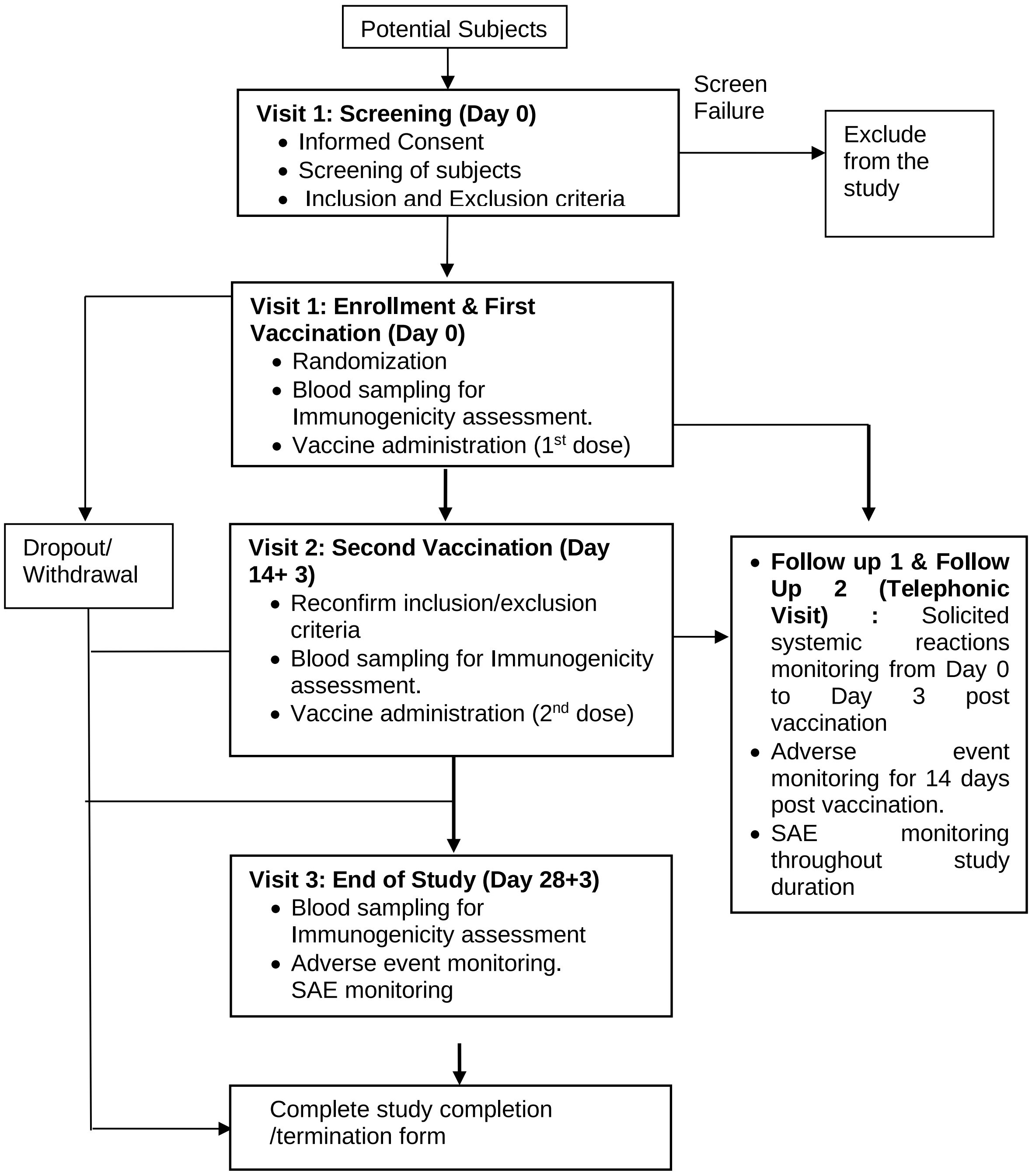

7.0 SELECTION AND WITHDRAWAL OF SUBJECTS

7.1 Inclusion Criteria

1. Individuals aged 1 year to 60 years both inclusive
2. Voluntary written informed consent form for study participation provided by an individual or his/her legally acceptable representative. Subjects aged 12 to <18 years, must sign an assent for the study and a parent or a legal guardian have to sign the informed consent. Subject aged 7 to 11 years need to provide an oral consent/assent and the consent form must be signed by parent/legally acceptable representative. Less than 7 years no need of assent, LAR can give consent.
3. An individual who can be followed up during the study period and is capable of complying with the study requirements.
4. Healthy subjects as determined by: Medical history, Physical examination and Clinical judgment of the investigator

7.2 Exclusion Criteria:

Any one of the following

1. History of cholera vaccinations or history of cholera.
2. History of hypersensitivity reactions to other preventative vaccinations.
3. Immune function disorders including immunodeficiency diseases.
4. 38°C or higher body temperature measured prior to investigational product dosing.
5. Diarrhea or abdominal pain lasting 2 weeks or longer within 6 months prior to study initiation. Also, administration of antidiarrheal drugs or antibiotics to treat diarrhea within 1 week prior to study initiation.
6. Abdominal pain, nausea, vomiting, or decreased appetite within 24 hours prior to study initiation.
7. Pregnant or lactating women.
8. Children vaccination within 1 month prior to study initiation or planned vaccination during the study.
9. Participation in another clinical trial with investigational product dosing within 6 months prior to study initiation.
10. An individual thought to have difficulty in participating in the study due to severe chronic diseases, based on the judgment of the investigator.

11. An individual thought to have difficulty participating in the study due to other reasons, based on the judgment of the investigator.
12. If female patient will get pregnant after first vaccination

7.3 Withdrawal of Subjects

Subjects will be discontinued or withdrawn from the study in case of following scenarios:

1. If the subject requests discontinuation or withdraws consent.
2. If the subject experiences a serious adverse event (SAE) which makes him/her incapable of further participation in the study.
3. Any entry criteria are violated, and the violation becomes apparent during the course of the study.
4. In case where the subject does not meet the inclusion and exclusion criteria, withdraws the informed consent or when a subject is required to be withdrawn solely at the discretion of investigator.
5. Subject does not report to the hospital for any of the 2 vaccination days or follow up visit beyond the allowable window period (+3 days) for blood withdrawal for the immunogenicity testing.
6. The subjects will be excluded from the second dose, If Life-threatening allergic reaction after a previous dose of any cholera vaccine or if female subject gets pregnant after first vaccination.
7. If there is a significant change in the vital signs, physical examination considering the safety of the subject.

In the event of withdrawal of a subject, the investigator shall assess the primary cause for the subject's withdrawal and document this in the Source and Case Report Form (CRF).

Subjects will be informed that they have the right to withdraw from the study at any time, without prejudice to their medical care, and that they are not obliged to state their reasons. Any withdrawal must be fully documented in the CRF and source documents. The investigator may also withdraw a subject at any time if this is considered to be in the subject's best interest.

Subjects withdrawn from the study will not be replaced.

8.0 STUDY DATA COLLECTION

8.1 Study Visits:

Following activities and assessments will be performed at each visit.

Visit 1: Screening, Enrollment and first Vaccination Visit (Day 1)

a. Subject 1 year to 60 years age will be screened for this study.

b. Written informed consent from subject/parents/legally accepted representative (LAR) will be obtained prior to performing any relative activity. Subject demography, medical history, prior medication and vaccination details, vital signs, physical examination will be done and results will be recorded in CRF pages.

c. If the subject meets the inclusion and none of the exclusion criteria, he/she will be enrolled, allocated to one of the study arms as per randomization envelopes

d. Urine Pregnancy Test for female patients who are of child bearing potential and sexually active.

e. A blood sample of 4 ml will be withdrawn (for antibody titers) before administration of first vaccine dose.

f. Subject will receive first dose of either Euvichol-Plus or Shanchol™ based upon the randomization in the Cohort 1 and 2. Concomitant Medication details will also be recorded.

g. Subjects will be observed at the study site for 30 minutes after vaccination. Examination for any systemic reactions will be made and findings will be recorded in the CRF.

h. Subject/parents/LAR of the subject will be instructed to evaluate systemic reactions and record the temperature at home for 3 days post-immunization (At day 0, 1, 2 and 3). Diary card will be issued for the filling the observations by the subject /parents/LAR

i. Schedule next site visit after 2 weeks. Subject/parents/LAR will be instructed for his/her subsequent appointment for immunization.

Visit 2: Second Vaccination Day 14 (+ 3 days after the first dose)

a. Any AE since the last vaccination will be recorded. Any unreported SAE since the last visit will also be recorded and reported by telephone/ facsimile/e-mail by the investigator as per regulatory requirements. Original Diary Card will be collected and data will be reconciled with the CRF.

b. Vital Signs, Physical and clinical examination will be done and results will be recorded in CRF pages. Concomitant Medication and vaccination details will also be recorded.

c. A blood sample of 4 ml will be withdrawn (for antibody titers) before administration of second vaccine dose.

d. If subjects are not excluded for the second dose of the vaccination as per the withdrawal criteria mentioned in section 7.3, subject will be administered orally second dose of either of the Test or Comparator vaccine as per the treatment allocated at visit 1 in the Cohort 1 and 2. Subjects will be observed at the study site for 30 minutes after immunization. Examination for any systemic reactions will be made and findings will be recorded in the CRF.

e. Subject/parents/LAR of the subject will be instructed to evaluate systemic reactions and record the temperature at home for 3 days post-immunization (At day 0, 1, 2 and 3). Diary card will be issued for the filling the observations by the subject/parents/LAR of the subject.

f. Schedule next site visit after 2 weeks. Subject/parents/LAR of the subject will be instructed for his/her subsequent appointment.

Follow up 1 & 2 (Telephonic Visits)

The appointed field officer will contact (email/phone/visit) the subject/parents/LAR after vaccination to determine the subject health status.

Visit 3: End of Study (EOS) visit Day 28 (+ 3 days after the second dose)

a. Any AE since the last vaccination will be recorded. Any unreported SAE since the last visit will also be recorded and reported by telephone/ facsimile/e-mail by the investigator as per regulatory requirement. Original Diary Card will be collected and data will be reconciled with the CRF.
b. Vital Signs, Physical and clinical examination will be done and results will be recorded in CRF pages. Concomitant Medication and vaccination details will also be recorded.
c. 4 ml of blood sample will be withdrawn for antibody titers.
d. Complete all pending information on the CRF including study completion page.

8.2 Method of blood collection and storage

Blood samples for antibody titers will be taken immediately before first and second vaccination and 2 weeks after the second dose of the study vaccine. Hence, 3 blood samples will be collected for each subject over the entire study period.

On each of the scheduled days, 4 ml of venous blood drawn from all the subjects will be collected in plain tubes. Clotted blood in the plain tube will be centrifuged to obtain serum. Serum obtained after centrifugation will be divided in two parts to be stored in two separate cryovial. At least 1.0 ml of the serum will be stored in one cryovial with the remaining serum transferred to the second cryovial. All cryovial containing serum for antibody analysis will be stored at or below - (minus) 20° C. One set of cryovial (containing 1.0 ml serum) will be sent to the central laboratory and the other will be stored at the site, which will be shipped to the sponsor on completion of the trial. The site laboratory assistant will be responsible for the labeling of the sera, labels will be provided by the sponsor. All serum samples will be sent to central laboratory with labels mentioning the protocol number, Enrollment ID of the subject, Subject initial and visit date (date of sample withdrawn).

8.3 Central Laboratory

Analysis will be done at the designated laboratory i.e NICED Kolkata.

8.4 Schedule of events

The summary of assessments performed during the trial is mentioned in Annexure –II.

9.0 PROTOCOL DEVIATIONS AND VIOLATIONS

A protocol deviation is defined as failure to fully comply with the final study protocol approved by the Ethics Committee (EC).

The deviation can be classified further as:

- Protocol Violation - Serious non-compliance with the protocol
- Protocol Deviation - Less serious non-compliance which needs adequate documentation

All violations or deviations from the approved protocol should be documented. The following details will be captured:

- Description of deviation
- Date of deviation
- Date reported and by whom
- Decision of sponsor
- Authorization of waiver, if applicable

All protocol violations or deviations will be reported to the sponsor company/CRO immediately to confirm subject continuation or withdrawal from the study. If the sponsor company agrees to the subject continuing in the study a note to file must be made by site personnel and the study monitor.

In case of a deviation, the investigator will inform CRO and seek approval for continuation/discontinuation of the concerned subject. The Investigator should not deviate from the protocol. The EC will be informed of protocol deviations by the investigator, according to applicable regulations and the EC's established procedures. The EC must provide written agreement for any deviation prior to continuing with the study. Unless CRO has consented to any such deviations in writing, CRO will not assume any resulting responsibility or liability. In medical emergencies, prior approval for protocol deviations will not be required.

10.0 ADVERSE EVENT

10.1 Adverse Event Monitoring

Any adverse events or side effects will be elicited by recording the response to a standard question "Have you noticed any problems with the treatment".

10.2 Adverse Event Documentation

Recording of AEs will begin after the ICD is signed by the subject or subject/parent/LAR. Any medical event occurring before the first dose of the vaccine will be regarded as a pre-treatment administration event. All events occurring after the first dose and prior events that worsen any time after the first dose will be regarded as Treatment Emergent Adverse Events (TEAE).

Any AE occurring after providing the informed consent and until the last study visit will be recorded on the CRF. All TEAEs occurring during the study period will be followed up by the investigator until resolution or stabilization of the event or until the subject is lost to follow-up. This follow-up should be documented in the subject medical records and may be extended beyond the end of the study period.

An **Adverse Event (AE)** is defined as any untoward medical occurrence (including a symptom/ disease or an abnormal laboratory finding) in a patient or clinical investigation subject administered a pharmaceutical product at any dose that does not necessarily have to have a causal relationship with this treatment. An adverse event can, therefore be any unfavorable and

unintended sign (including an abnormal laboratory finding, for example), symptom, or disease temporally associated with the use of an investigational product, whether or not considered related to the investigational product. This definition includes inter-current illnesses or injuries and exacerbation of pre-existing conditions.

Adverse Event Following Immunization (AEFI): This is defined as any untoward medical occurrence which follows immunization and which does not necessarily have a causal relationship with the use of the vaccine. The adverse event may be any unfavorable or unintended sign, an abnormal laboratory finding, a symptom or a disease.

The adverse events reported and as observed by the investigator will be filled in the case report form and maintained for the purpose of documentation.

The investigator must report adverse events which are clinically apparent, or in the investigator's opinion clinically significant, in the relevant part of the case report form.

Systemic Reactions (Protocol specific)

- All reaction during the study will be inquired & recorded for up to 30 minutes on the day of each vaccination and for 3 days post-vaccination. Systemic reactions[7, 8] (fever, Headache, acute allergic reaction, Loss of Appetite, fatigue, vomiting and diarrhea) will be recorded.
- Solicited reactions[12, 13] will be graded as follows.

**General reaction**	**Mild (Grade 1)**	**Moderate (Grade 2)**	**Severe (Grade 3)**	**Potentially Life Threatening (Grade 4)**
Fever	38.0-38.4°C	38.5-38.9°C	39 – 40	> 40.0°C
Headache	No interference with activity	Repeated use of non- narcotic pain reliever > 24 hours or some interference with activity	Significant; any use of narcotic pain reliever or prevents daily activity	Emergency room (ER) visit or hospitalization
Fatigue	No interference with activity	Some interference with activity	Significant; prevents daily activity	ER visit or hospitalization
Myalgia	No interference with activity	Some interference with activity	Significant; prevents daily activity	ER visit or hospitalization
Vomiting/ Nausea	No interference with activity or 1 – 2 episodes/24 hours	Some interference with activity or > 2 episodes/24 hours	Prevents daily activity, requires outpatient IV hydration	ER visit or hospitalization for hypotensive shock

General reaction	Mild (Grade 1)	Moderate (Grade 2)	Severe (Grade 3)	Potentially Life Threatening (Grade 4)
Diarrhea	2 – 3 loose stools or < 400 gms/24 hours	4 – 5 stools or 400 – 800 gms/24 hours	6 or more watery stools or > 800gms/24 hours or requires outpatient IV hydration	ER visit or hospitalizatio n

The occurrence of solicited systemic reactions will be noted in subject diary card by the parent/LAR and copied on to relevant section of the CRF pages by the investigator/designee at the subsequent clinic visit. These reactions will be recorded on the "Solicited Systemic Reactions" section of the CRF along with 'Date of resolution'; if they do not resolve satisfactorily by 3 days following study immunization.

Unsolicited Adverse Events

An unsolicited adverse event is an observed adverse event that follows immunization but does not fulfill the conditions prelisted in the patient diary in terms of symptom/sign and/or timing of onset post-vaccination. All such events will be recorded in the 'Adverse event' pages in the CRF.

10.3 Severity and Relation of Adverse Event

All solicited systemic reactions and unsolicited adverse events observed or reported/volunteered by subject/parent/LAR must be recorded in the CRFs with information about severity (i.e., whether mild, moderate, severe) and possible relation to the study vaccine.

Mild: usually transient in nature and generally not interfering with normal activities

Moderate**:** sufficiently discomforting to interfere with normal activities

Severe: prevents normal activities

Relationship of AE to study medication will be described as[14]:

1. **Case with adequate information for causality conclusion**

   A case with adequate information for causality conclusion can be classified as follows:

   A. **Consistent causal association to immunization**

      A1. Vaccine product-related reaction; or

      A2. Vaccine quality defect-related reaction; or

      A3. Immunization error-related reaction; or

      A4. Immunization anxiety-related reaction.

   B. **Indeterminate**

      B1. Temporal relationship is consistent but there is insufficient definitive evidence that vaccine caused the event (it may be a new vaccine-linked event). This is a potential signal and needs to be considered for further investigation.

B2. Reviewing factors result in conflicting trends of consistency and inconsistency

With causal association to immunization (i.e. it may be vaccine-associated as well as coincidental and it is not possible clearly to favors one or the other).

C. **Inconsistent causal association to immunization (coincidental)**: This could be due to underlying or emerging condition(s) or conditions caused by exposure to something other than vaccine.

2. **Case without adequate information for causality conclusion**

This case is categorized as "unclassifiable" and requires additional information for further review of causality. The available information on unclassifiable cases should be placed in source document which should be periodically reviewed to see if additional information is available for classification.

The case should be classified and the classification documented in CRF if this information is available during the AE follow-up period as defined in section 13.2.

Factors that would assist in determining the Causality include:

1. Timing of occurrence of the adverse event
2. Absence of symptoms related to the event prior to exposure
3. Consistency of the event with the established pharmacological/toxicological effects of the product
4. Consistency of the event with the known effects of other products in the class
5. Supporting evidence from other studies or absence of alternative explanations

The serious adverse drug reaction will be recorded in the Serious Adverse Event Reporting form provided separately.

10.4 Serious Adverse Events Reporting

Investigator shall report all serious and unexpected adverse events, to the Licensing Authority, the Sponsor, and the Ethics Committee that accorded approval to the study protocol, within 24 hours of their occurrence as per Table 5 of the New Clinical Trial rules March 2019 by telephone/ facsimile/e-mail. The report of the serious adverse event of death, after due analysis shall be forwarded by the Investigator to Chairman of the Ethics Committee and Chairman of the Expert Committee constituted by the Licensing

Authority under Table 5 of the New Clinical Trial rules March 2019 with a copy of the report to the Licensing Authority and the head of the Institution where the trial has been conducted within 14 days of the knowledge of occurrence of SAE death. The report of the serious adverse event other than death, after due analysis shall be forwarded to the Licensing Authority, Chairman of the Ethics Committee and the head of the Institution where the trial has been conducted within 14 days of the reporting of serious adverse event.

For the purpose of this technical protocol, a serious adverse event is defined as any unfavorable medical occurrence that

- Results in the death of the participant,
- Is life-threatening,

Note: The term "life-threatening" refers to an event in which the patient was at risk of death at the time of the event; it does not refer to an event, which hypothetically might have caused death if it were more severe.

- Requires inpatient hospitalization or prolongation of existing hospitalization.
- Results in persistent or significant disability or incapacity,

10.5 Follow up of subject with Adverse Events

Subjects with adverse events shall be followed up till the resolution of the adverse event or last scheduled follow up visit, whichever is earlier. Serious adverse events shall be followed up till their resolution or stabilization with reasonable background of clinical & scientific judgment.

Irrespective of the investigator's statutory obligations, the sponsor will report all pharmacovigilance data to the competent authorities and to all investigators involved in accordance with requirements of the new drug and new clinical trial rules March 2019, Guidelines for Good Clinical Practice and local regulatory requirements.

10.6 Precautions

In case of any serious drug related adverse event the investigator will contact the sponsor immediately:

Techinvention Lifecare Pvt Ltd.

Name of the person to be contacted: Mr. Syed S Ahmed

Contact no:9867639480 Email ID: syed@techinvention.biz

11.0 DATA AND SAFETY MONITORING BOARD

Data and Safety Monitoring Board (DSMB) will be constituted to review the safety data of the study. It will consist of experts in Medical Science, biostatistics, and clinical studies. DSMB will review the 14 days post vaccination safety data of the subjects of cohort 1 and accordingly give recommendation for the enrollment of cohort 2. DSMB will also review the 14 days post vaccination safety data of the subjects of cohort 2. Subsequent DSMB meeting will be decided as required. Prior to DSMB meeting, the safety data will be submitted to DSMB by sponsor in a format acceptable to the Board.

12.0 STATISTICAL METHODS

Data obtained from the study subjects will be analyzed in three forms; safety set, Intention to Treat (ITT) set, and Per Protocol (PP) set.

The safety set includes all subjects who participated in the study and had at least one dose of the Investigational product, based on which safety will be assessed. The ITT set includes subjects who had at least one measurement of anti-V. Cholera O1 Ogawa, Inaba and O139 antibody titer after investigational product dosing, based on which immunogenicity will be assessed. The PP set includes ITT subjects who completed this study per protocol, based on which immunogenicity will be assessed additionally.

A p-value of less than 0.05 will be considered the threshold to claim statistical significance.

13.0 ETHICAL AND REGULATORY CONSIDERATIONS

The proposed clinical study will be conducted after the approval of Institutional Review Board/ Ethical committee.

13.1 GCP and Monitoring

The study will be conducted according to India new regulatory guidelines, declaration of Helsinki and Good Clinical Practices. Regular monitoring will be done by the representatives of the sponsor periodically.

13.2 Ethics Committee Approval

The investigator will submit the appropriate documents to the Institutional Review Board / Ethics Committee (registered with DCGI) for approval prior to enrolment of subjects. Details of the methods to be used for recruitment of subjects into the study should also be provided. If, during the study, it is necessary to amend either the protocol or the informed consent form, the investigator will be responsible for ensuring the ethics committee reviews and approval of these amended documents. Apart from EC approval, the trial will be registered in Clinical Trial Registry India before the first subject enrolment.

13.3 Protocol Amendments

Proposed amendments to the protocol and aforementioned documents must be submitted to the sponsor for review and approval, and then to the EC and the regulatory authority as necessary. Amendments may be implemented only after a copy of the EC approval letter has been transmitted to the sponsor. Amendments that are intended to eliminate an apparent immediate hazard to subjects may be implemented prior to receiving sponsor or EC approval. However, in this case, approval must be obtained as soon as possible after implementation. Also, in that event, the Investigator must notify the EC in writing within five (5) working days after the implementation.

13.4 Written Informed Consent and Subject Information

The purpose of the study, the procedures to be carried out and potential risks/benefits will be described to study subject/parent/LAR in non-technical terms. Study subject/parent/LAR will be required to read, understand and sign a consent form prior to the inclusion in study and will be assured that they can withdraw from the study at any time. In case of assent age group from 7 to 11 need to give verbal assent. 12 to <18 years need to give written assent. Less than 7 years no need of assent, LAR can give consent.

Parent/LAR who is asked for their child's participation in clinical research is entitled to choose whether or not to take part. Their decision is voluntary and they should be competent to understand what is involved. Consent forms are designed to assure the protection of subject's rights. Subject must receive adequate verbal and written information in their own language. Either the investigator or medically qualified personnel who are the part of trial team will perform the verbal explanation to the subject/parent/LAR. The verbal explanation will cover all the elements specified in the written information provided for the subject. The investigator will inform the subject/parent/LAR of the aims, methods, anticipated benefits and potential hazards of the study including any discomfort it may entail. The subject/parent/ LAR must be given every opportunity to clarify any points he or she does not understand and if necessary, ask for more information. At the end of the discussion subject/parent/LAR may be given time and then asked to reflect his/her opinion. It should be emphasized that subject are at liberty to withdraw their consent for their's participation at any time, without penalty or loss of benefits to which they are otherwise entitled. The investigator is responsible for obtaining the subject freely given consent. The written consent form provided to the subject /parent/LAR should be signed and dated by the subject/parent/LAR as well as the investigator. Subject should be given

a copy of the document, which includes the name and phone number of the person to contact in case of an emergency. The consent must be kept on file by the investigator for possible inspection, monitoring and audit by regulatory authorities and/or sponsor professional persons. The signature confirms the consent, which is based on information that has been understood.

In case the subject/parent/LAR is illiterate all the information will be explained to him/her in the presence of an impartial witness (a literate person who is not related to the study) who will also sign the ICD along with thumb impression of the subject/parent/LAR.

14.0 DATA AND SAFETY MONITORING BOARD [DSMB]

Independent oversight of this study will be provided by a DSMB, a multidisciplinary group of expertise in the fields of epidemiology and community medicine, drug safety and statistics. The DSMB consists of a minimum of three members who have no direct role in the conduct of this clinical trial, or any other conflict of interest, and they operate based on a study specific DSMB Charter. The DSMB will review interim and cumulative safety data. The DSMB may recommend discontinuation of the study if they find evidence of unacceptable risk to patients. SPONSOR will have the responsibility for the final decision regarding the DSMB-recommended course of action. The composition and the DSMB meeting schedule will be included in the DSMB charter.

15.0 DATA HANDLING

Any data collected on each subject who is enrolled in the study must be noted in the CRF. There should be a complete and accurate record of the subject’s data collected during the study. There must be an explanation if it is not possible to collect certain data or if it is missing. The investigator at each site is responsible for the quality of the data recorded in the CRF, and has to sign and date CRF pages after completion as per instruction provided.

16.0 ACCESS TO SOURCE DATA/DOCUMENTS

All trial related documents should be stored in a proper place. Any or all of the study related documents may be subject to, and should be available for monitoring/audit by sponsor’s monitor/ auditor and inspection by the IRB/IEC or regulatory authorities.

17.0 RECORD KEEPING AND ARCHIVING OF DATA

In accordance with applicable regulatory requirements, following the closure of the study, the investigator will maintain a copy of all site study records in a safe and secure location for retaining these records for 15 years minimum.

18.0 STUDY MONITORING

The sponsor or sponsor’s representative will maintain a close liaison with the investigators and staff to clarify problems that may arise during the study, and to ensure that the investigation is being carried out according to the protocol. The study will be monitored throughout by the responsible monitor in order to maintain current and personal knowledge of the study through review of the records, comparison with source documents, observation and discussion of the conduct and the progress of the study. The monitoring will consist of personal visits before the study is initiated, when the center is initiated, at appropriate intervals during the study and at the end of the study. The monitoring will also include communications via telephone and letter.

The sponsor is responsible according to new drug and new clinical trial rules march 2019, GCP guidelines for assuring proper study conduct with regard to protocol adherence and validity of the data recorded on the CRFs. The monitor of sponsor or sponsor representative will assist the investigator in the maintenance of complete, legible, well-organized, and easily retrievable data. In addition, the monitor will ensure that the investigator understands all applicable regulations concerning the clinical evaluation of an investigational drug, as laid down in New drug and new clinical trial rules and GCP guidelines.

The investigator agrees to allow the monitor access to the study drug dispensing and storage area and to all clinical data of the study subjects for the above purposes and agrees to assist the monitor in these activities. The investigator accepts that the monitor will visit the site at regular intervals to review and verify the data collected. The monitor will regard all information, which is supplied to him or her as strictly confidential. The monitoring visits are for the purpose of verifying adherence to the protocol and for completeness and exactness of data entered in the CRFs and drug inventory forms. The monitor will verify CRF entries by comparing them with the source data, which will be made available for this purpose. The monitor may retrieve complete sections from CRF at each visit. Adequate time and space for these visits should be made available by the investigator.

19.0 QUALITY CONTROL AND QUALITY ASSURANCE

The study may be subject to audit by sponsor, or external auditor or regulatory authority. In such case, all relevant information must be made available by the investigator and he/she, as well as the involved site personnel, must reserve time for review and discussions of any findings during the audit.

20.0 MAINTENANCE OF LOGS, MONITORING AND AUDIT PLAN

The investigator and the staff should maintain the Site Master File, logs concerning the clinical trial and source documents.
Complete monitoring and audit details will be provided in advance by the sponsor monitors to the investigator and the staff.

19.1 INFORMATION DISCLOSURE AND INVENTIONS

19.2 Ownership

All data and records provided by sponsor or generated during the study (other than a subject's medical records) and all inventions discovered in the course of conducting the study are the property of Techinvention. No amendment to the protocol can be made without prior discussion/approval with sponsor.

19.3 Confidentiality

The investigator and other study personnel will keep confidential any information provided by sponsor. (Including this protocol) related to this study and all data and records generated in the course of conducting the study, and will not use the information, data, or records for any purpose other than conducting the study. These restrictions do not apply to:

- Information, which becomes publicly available through no fault of the investigator or study site personnel
- Information, which it is necessary to disclose in confidence to the ethics committee solely for the evaluation of the study

- Information, which it is necessary to disclose in order to provide appropriate medical care to a study subject
- Study results, which may be published as described in the next section.

21.0 PUBLICATION POLICY

The investigator agrees that information developed during the course of this clinical study will be published in agreement with sponsor. In accordance with generally recognized principles of scientific collaboration, co-authorship with sponsor. Personnel will be discussed and mutually agreed upon before submission of a manuscript to a publisher. Prior to submitting for publication, presenting, using for instructional purposes or otherwise disclosing the results of the study, the investigator shall allow sponsor a period of at least thirty (30) days [or, for abstracts, at least five (5) working days] to review the proposed publication or disclosure prior to its submission for publication or other disclosure. Publications or disclosures of study results shall not include other confidential information of sponsor.

All data presentation by each investigator has to be done with consent of the sponsor.

22.0 STUDY TERMINATION AND SITE CLOSURE

Upon completion of the study, the monitor will conduct the following activities in conjunction with the investigator or site staff, as appropriate, including but not limited to the following:

- Return of all study data to the sponsor.
- Data queries.
- Review of site study records for completeness.
- Investigational product compliance

In addition, the sponsor reserves the right to temporarily suspend or prematurely discontinue this study at any time for reasons including, but not limited to, ethical issues, safety issues or severe non-compliance. If the sponsor determines such action is needed, the sponsor will discuss this with the investigator (including the reasons for taking such action) at that time. When feasible, the sponsor will provide advance notification to the investigator of the impending action prior to it taking effect.

The sponsor will also inform the regulatory authorities of the suspension or termination of the study and the reason(s) for the action. The investigator must inform the subjects and the EC promptly and provide the reason for the suspension or termination.

If the study is prematurely discontinued, all study data must be returned to sponsor.

23.0 FINANCING AND INSURANCE

Sponsor will take insurance for clinical trial . This insurance will provide coverage for medical care in the event an AE occurs.
1. In the event of an injury, Sponsor shall provide free medical management as long as required.
2. In the event of a trial related injury or death, the Sponsor shall provide financial compensation for the injury or death.

24.0 AMENDMENT TO THE PROTOCOL

Any significant change in the study procedure or study design will only be effective upon mutual agreement with the Sponsor, Investigator and Study Director. All such changes will be

documented in the amended version of the protocol and a list of changes with reference to the previous version will be generated and documented.

25.0 REFERENCES

1. Ali M et al. Updated global burden of cholera in endemic countries. PLoS Negl Trop Dis 2015;9(6):e0003832.
2. Barua D. History of cholera. In: Cholera. Springer; 1992. p. 1–36.
3. Akanda AS, Jutla AS, Islam S. Dual peak cholera transmission in Bengal Delta: a hydroclimatological explanation. Geophys Res Lett 2009;36(19).
4. Bharati K, Bhattacharya SK. Cholera outbreaks in South-East Asia. In: Cholera outbreak. p. 87–116.
5. Kanungo S et al. Cholera in India: an analysis of reports, 1997–2006. Bulletin of the World Health Organization; 2010. p. 185–91.
6. WHA64, R., 15. Cholera: mechanism for control and prevention. Sixty-fourth World Health Assembly, Geneva, 16, 2011. 1.
7. Bhattacharya SK et al. 5 year efficacy of a bivalent killed whole-cell oral cholera vaccine in Kolkata, India: a cluster-randomised, double-blind, placebo controlled trial. Lancet Infect Dis 2013;13(12):1050–6.
8. Kanungo S et al. Immune responses following one and two doses of the reformulated, bivalent, killed, whole-cell, oral cholera vaccine among adults and children in Kolkata, India: a randomized, placebo-controlled trial. Vaccine 2009; 27(49):6887–93.
9. Kanungo S et al. An open label non-inferiority trial assessing vibriocidal response of a killed bivalent oral cholera vaccine regimen following a five year interval in Kolkata, India. PLoS Negl Trop Dis 2015;9(5):e0003809.
10. Organization, W.H., Cholera vaccines: WHO position paper–August 2017. Weekly Epidemiological Record, 2017. 92(34): p. 477–498.
11. Kanungo S et al. Flexibility of oral cholera vaccine dosing—a randomized controlled trial measuring immune responses following alternative vaccination schedules in a cholera hyper-endemic zone. PLoS Negl Trop Dis 2015;9(3):e0003574.
12. Marcy SM, Kohl KS, Dagan R, Nalin D, Blum M, Jones MC, Hansen J , Labadie J, Lee L, Martin BL, O'Brien K, Rothstein E, Vermeer P, The Brighton Collaboration Fever Working Group. Fever as an adverse event following immunization: case definition and guidelines of data collection, analysis, and presentation. Vaccine 2004; 22:551–556
13. Toxicity Grading Scale for healthy Adults and Adolescent Volunteers Enrolled in Preventive Vaccine Trials, U.S. Department of Health and Human Services, Food
14. CAUSALITY ASSESSMENT OF AN ADVERSE EVENT FOLLOWING IMMUNIZATION (AEFI), User manual for the revised WHO classification. WHO/HIS/EMP/QSS. MARCH 2013.
15. Mohammad Ali, Anna Lena Lopez et all; The global burden of cholera; Bulletin of the World Health Organization, 2012 Mar 1, 90(3);209-218A
16. Venkata Raghava Mohan, Santosh Raj, et all; Safety and immunogenicity of a killed bivalent (O1 and O139) whole-cell oral cholera vaccine in adults and children in Vellore, South India; PLOS ONE | https://doi.org/10.1371/journal.pone.0218033 June 18, 2019

17. Young Ok Baik,1 Seuk Keun Choi et all; Safety and Immunogenicity Assessment of an Oral Cholera Vaccine through Phase I Clinical Trial in Korea; Korean Med Sci 2014; 29: 494-501; http://dx.doi.org/10.3346/jkms.2014.29.4.494
18. Baik YO, Choi SK et all; A randomized, non-inferiority trial comparing two bivalent killed, whole cell, oral cholera vaccines (Euvichol-Plus vs Shanchol™) in the Philippines. Vaccine. 2015 Nov 17;33(46):6360-5. doi: 10.1016/j.vaccine.2015.08.075. Epub 2015 Sep 5.
19. Organization, W.H., Cholera vaccines: WHO position paper–August 2017. Weekly Epidemiological Record, 2017. 92(34): p. 477–498.
20. Odevall L, Hong D, Digilio L, et al. The Euvichol-Plus story - Development and licensure of a safe, effective and affordable oral cholera vaccine through global public private partnerships. Vaccine. 2018;36(45):6606–6614. doi:10.1016/j.vaccine.2018.09.026
21. Baik Y.O., Choi S.K., Kim J.W., Yang J.S., Kim I.Y., Kim C.W. Safety and immunogenicity assessment of an oral cholera vaccine through phase I clinical trial in Korea. J Korean Med Sci. 2014;29(4):494–501. [PMC free article] [PubMed] [Google Scholar]
22. Baik Y.O., Choi S.K., Olveda R.M., Espos R.A., Ligsay A.D., Montellano M.B. A randomized, non-inferiority trial comparing two bivalent killed, whole cell, oral cholera vaccines (Euvichol-Plus vs Shanchol™) in the Philippines. Vaccine. 2015;33(46):6360–6365. [PubMed] [Google Scholar]
23. WHO. Out of cold chain (OCC) and Controlled Temperature Chain (CTC) use of vaccines. Immunization Practices Advisory Committee; 2017 [updated 2017; cited]; http://www.who.int/immunization/programmes_systems/supply_chain/ctc/en/.
24. WHO. WHO Prequalified Vaccines [cited]; Available from: https://extranet.who.int/gavi/PQ_Web/PreviewVaccine.aspx?nav=0&ID=249.

26.0 ANNEXURE

- Annexure I: INVESTIGATOR LIST
- Annexure II: DECLARATION OF HELSINKI
- Annexure III: SCHEDULE OF EVENTS

File No: BIO/CT/20/000135

Government of India
Directorate General of Health Services
Central Drugs Standard Control Organization
(Biological Division)

FDA Bhawan Kotla Road,
New Delhi-110002
Date:
14 JUN 2022

To

M/s Techinvention Lifecare Pvt Ltd,
702, Samarpan Complex, New Link Road Chakala,
Andheri East, Mumbai (India) – 400099.

Subject: Clinical site addition for conducting a clinical trial titled "A Phase III, open label, multicenter, parallel group, randomized Clinical Study to compare the Immunogenicity and Safety of Euvichol-Plus vaccine with Oral Cholera Vaccine Shanchol^{TM} in healthy adults and children above age of one year" [Protocol: EPV/TLC/P-III/2020, Version No.:2.0, Date: 7th March 2021]-regarding.

Reference:

1. Your Letter No. nil dated 23.03.2022 submitted to this office vide diary no: 2743 dated 01.04.2022.
2. Your Letter No. TI/RA/07 dated 04.10.2021 submitted to this office vide diary no: 9510 dated 07.10.2021.
3. Permission in Form CT-06 vide CT.no: 13/2021 dated 20.05.2021 issued from this office.

Sir,

With reference to subject cited above and in continuation to permission granted vide (ref.2) for conduct of Phase-III clinical trial of Oral Cholera Vaccine (Protocol No: EPV/TLC/P-III/2020, Version No.:2.0, Date: 7th March 2021), based on information/documents submitted, this Directorate acknowledges addition of following clinical trial site for said study.

Name of clinical trial site	Ethics Committee Details	Principal Investigator
Aatman Hospital, 5 Anveshan Row House, Bopal Gram BRTS, Bopal-Ghuma Road, Bopal, Ahmedabad-380058, Gujarat, India.	Institutional Ethics Committee, 5 Anveshan Row House, Bopal Gram BRTS, Bopal- Ghuma Road, Bopal,Ahmedabad-380058, Gujarat, India. [ECR/1565/Inst/GJ/2021]	Dr. Chintan B Patel.

However, all other terms & conditions as stipulated in the said clinical trial permission shall remain unchanged.

Yours faithfully,

(Dr. S. Eswara Reddy)
Joint Drugs Controller (India)
